# Mapping Charge Interactions in Intrinsically Disordered Proteins

**DOI:** 10.1002/advs.202514056

**Published:** 2025-11-26

**Authors:** Michael Phillips, Andrea Holla, Magdalena Wojtas, Aritra Chowdhury, Andrea Sottini, Sebastian L. B. König, Natalie Mutter, Nick Lamb, Jonathan Huihui, Monika Lopko, Andrea Soranno, Daniel Nettels, Andrzej Ożyhar, Benjamin Schuler, Kingshuk Ghosh

**Affiliations:** ^1^ Department of Physics and Astronomy University of Denver Denver CO 80208 USA; ^2^ Department of Biochemistry University of Zurich Zurich 8057 Switzerland; ^3^ Department of Biochemistry Molecular Biology and Biotechnology Wrocław University of Technology Wrocław 50‐373 Poland; ^4^ Department of Biochemistry and Molecular Biophysics, Center for Biomolecular Condensates Washington University in St. Louis St. Louis MO 63110 USA; ^5^ Department of Biochemistry and Department of Physics University of Zurich Zurich 8057 Switzerland

**Keywords:** intrinsically disordered proteins, single‐molecule FRET, polymer theory, polyelectrolytes

## Abstract

Intrinsically disordered proteins (IDPs) are often rich in charged residues, and electrostatic interactions have a pronounced effect on their conformational distributions, interactions and functions. However, attaining quantitative understanding of electrostatics is challenging because of the sequence‐specific arrangement of charges in the chain, the long‐range nature of electrostatic interactions, charge screening, and the condensation of counterions—effects that all need to be taken into account self‐consistently. Here, analytically tractable quantitative models are developed to predict ensemble average distances between any pair of residues in IDPs as a function of sequence and salt concentration, explicitly considering charge patterning. These models are tested systematically against extensive single‐molecule Förster resonance energy transfer (FRET) data mapping intrachain distances for a range of charged IDPs with different sequence compositions, as a function of salt concentration, and with different labeling positions and fluorophores. The resulting polymer model with a minimal set of adjustable parameters accounts for counterion condensation, the resulting effective charges, as well as dipolar interactions, and can be used to predict detailed intrachain distance maps between all residues. Analytical models of this kind offer a valuable complement to simulations and can provide fundamental insight into the interactions underlying the conformational distributions of IDPs.

## Graphical abstract

This study presents analytical models to predict intrachain distances in intrinsically disordered proteins (IDPs), accounting for charge patterning, salt concentration, and electrostatic effects. Validated against single‐molecule FRET data, a minimal‐parameter polymer model is identified that captures key interactions and chain dimensions. These models enhance the understanding of IDP behavior and complement computational simulations.

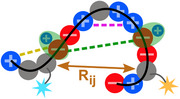

## 1 Introduction

Intrinsically disordered proteins (IDPs) are enriched in charged amino acids,^[^ ^1^ ^]^ and electrostatics thus play a key role in modulating their conformations and functions. Several aspects of electrostatics can affect IDP behavior. First, the number, type (positive or negative), and position of charges within the sequence influence the conformational ensembles of IDPs.^[^ ^2^, ^3^, ^4^, ^5^, ^6^, ^7^, ^8^, ^9^, ^10^, ^11^, ^12^, ^13^, ^14^, ^15^, ^16^, ^17^, ^18^ ^]^ Second, changes in salt concentration can screen electrostatics (attractive or repulsive) in a sequence‐dependent manner.^[^ ^10^, ^19^ ^]^ For example, screening the charge repulsion in polyelectrolytes leads to chain compaction, whereas screening charge attraction in polyampholytes can lead to chain expansion,^[^ ^3^, ^20^, ^21^, ^22^, ^23^ ^]^ and two sequences with the same charge composition but different charge patterning can exhibit markedly different response under identical changes in ionic strength.^[^ ^19^, ^24^ ^]^ Third, the effective charges in the polypeptide chain can be variable, contrary to the typical assumption that the side chains are fully ionized at neutral pH. The origin of this variable charge state is that ionizable residues are in an equilibrium between two states: fully ionized and complexed with oppositely charged ions.^[^ ^22^, ^25^, ^26^, ^27^, ^28^ ^]^ This phenomenon is often termed charge regulation for protonation equilibria, or charge renormalization for the interaction with other counterions from salt in solution. In both cases, the net result is that the effective charge of ionizable residues in an IDP can differ from the nominal charge commonly assigned to them.^[^ ^27^, ^28^, ^29^, ^30^, ^31^, ^32^ ^]^ The difference, often quantified by an effective degree of ionization, will depend on additional factors, such as the presence of other charges in the sequence, the dielectric constant in the vicinity of the chain, solution conditions (e.g., pH, ionic strength), and also on the conformation of the chain, which in turn depends on the sequence and degree of ionization. To answer the question of how these factors are coupled and how they influence IDP conformations, we need an approach integrating quantitative measurements and models.

**Figure 1:**
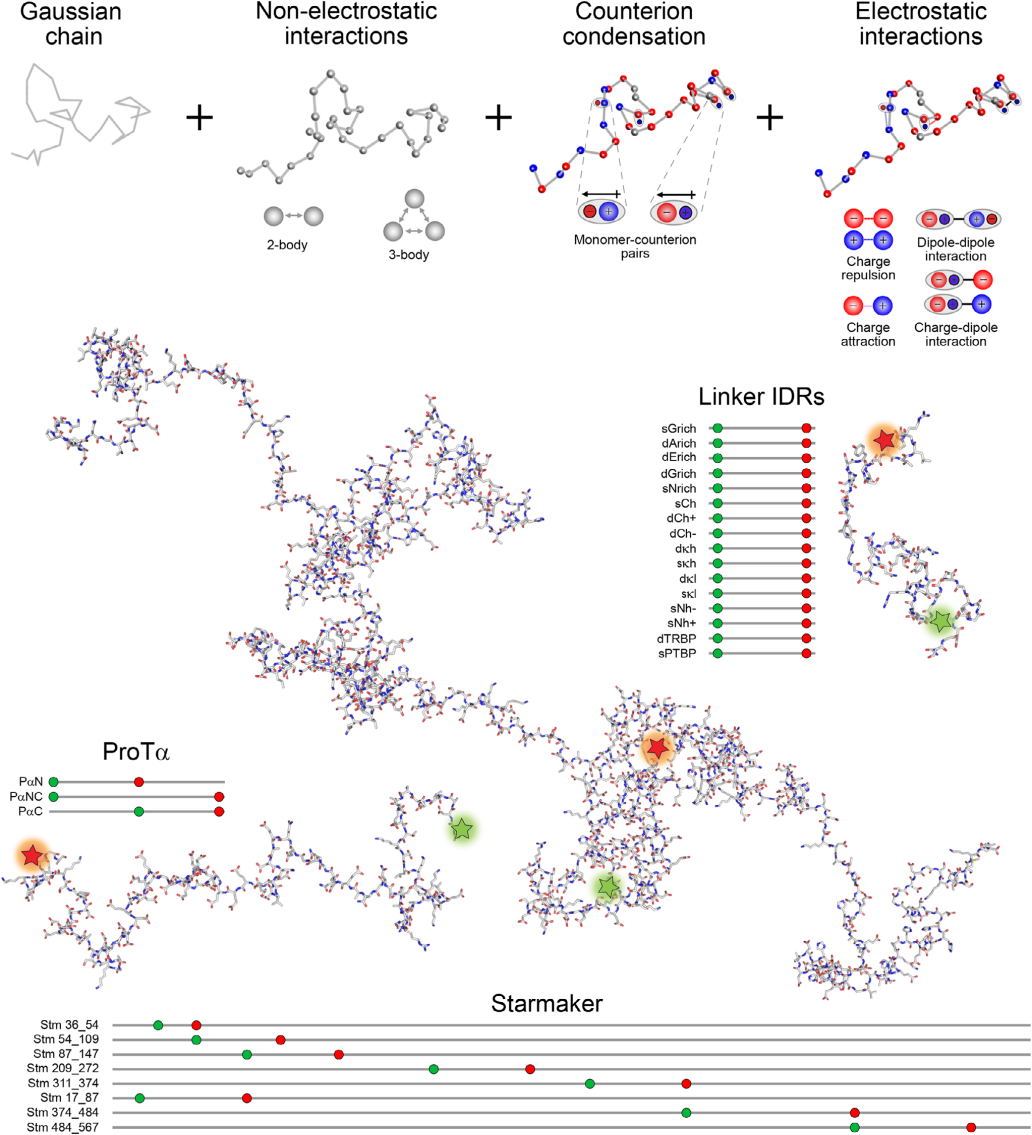
Illustration of the key components of the analytical theory used (top) and of the molecular systems investigated with single‐molecule FRET (bottom) in an integrated approach to quantify ion condensation and its impact on the chain dimensions. In the theory, Gaussian chain statistics are combined with two‐body and three‐body excluded volume interactions. Condensed counterions on the charged side chains form dipoles (shaded ellipses) and give rise to charge‐dipole, dipole‐dipole, and charge‐charge interactions. The theory accounts for these interactions for arbitrary sequences of positive (blue), negative (red) and neutral (grey) amino acids to predict ensemble‐averaged distances ($\left\langle R_{ij}^{2}\right\rangle$) between any residues *i*, *j*. A wide range of protein variants were used to parameterize the theory and test its predictions based on single‐molecule FRET measurements probing intrachain distances. Structural representations of the different disordered proteins and schematics of the sequences and labeling positions (red and green) are shown. The structural representations were generated from the sequences using AlphaFold 3^[^ ^41^ ^]^ and Afflecto.^[^ ^42^ ^]^ The naming of the different linker IDRs reflects their sequence properties (see Table S1, Supporting Information, and Holla et al.^[^ ^36^ ^]^ for details).

On the experimental side (see Sections S1 and S2, Supporting Information), we used single‐molecule Förster resonance energy transfer (FRET)^[^ ^33^, ^34^, ^35^ ^]^ to measure end‐to‐end and intrachain distances across 18 different disordered protein sequences with different compositions and patterning that cover a diverse sequence space (**Figure** **1**). A set of 16 naturally occurring intrinsically disordered regions (IDRs) of identical length but with large differences in amino acid composition, hydrophobicity, and charge content and patterning was previously selected from linkers found in RNA‐binding proteins,^[^ ^36^ ^]^ for which we probed the end‐to‐end distance (Table S1, Supporting Information). In a complementary set of samples, FRET dyes were placed at different positions in the highly negatively charged IDPs Starmaker (Stm)^[^ ^37^ ^]^ and Prothymosin α (ProTα)^[^ ^3^, ^38^ ^]^ to probe intra‐chain distances for different segments and segment lengths within those sequences (Figure 1; Table S1, Supporting Information). In addition to using this diverse set of sequences, we modulated the electrostatic interactions by probing the chain dimensions and intrachain distances across a broad range of concentrations of monovalent salt. The resulting data set thus provides rich information on electrostatic and non‐electrostatic interactions in disordered proteins across a wide range of sequence compositions, chain lengths, and salt concentrations, making it ideally suited for developing and testing a quantitative framework that describes charge interactions in IDPs.

Toward this aim, we developed analytically tractable quantitative models based on the Edwards–Muthukumar Hamiltonian^[^ ^39^, ^40^ ^]^ to predict ensemble average distances between any pair of residues in IDPs as a function of sequence, explicitly considering charge patterning and ion condensation. Our theory includes sequence‐specific electrostatic interactions between charges, as well as dipolar interactions (charge–dipole, and dipole–dipole) arising from ion‐pairs formed between charges on the polypeptide chain and condensed counterions (Figure 1). These models were then optimized and tested against the large set of single‐molecule FRET data. An advantage of analytical models is the ability to test hypotheses by varying the complexity of the models. Starting with the most complex model and subsequently reducing model complexity, we find that it is possible to describe the data by a minimal, near‐predictive model. In this framework, sequence‐specific non‐electrostatic interactions are quantified from measurements at high salt concentration, where electrostatics are fully screened. Counterion interactions are described by two parameters: an effective separation (dipole length) between the side chain charge and condensed ion, and an effective dielectric in the vicinity of the chain. Both parameters can be learned from the data and used in a transferable manner to describe the majority of the sequences. In this formalism, effective ionization is due to counterion condensation and is highly dependent on the ionic strength and on the protein sequence. Taken together, our study provides a stringent test of models of IDPs with different charge content, patterning, and screening based on experimental data, which enable a quantitative description of the critical role charge interactions play in the conformations of IDPs.

## 2 Model

### 2.1 Models for Calculating the End‐to‐End Distance

We developed an analytical model of an IDP with *N* residues, and *N*_+_ positive and *N*_−_ negative charges (upon full ionization). To model charge variation of residues, we assume that each charge type is ionized with a probability *α*_±_ ∈ [0, 1], yielding the effective charge *q*_*m*_ = +*α*_+_, *q*_*m*_ = −*α*_−_, or *q*_*m*_ = 0, depending on its classification (basic/acidic/neutral). We assume that charges of the same type (i.e., acidic or basic) have the same effective charge, irrespective of their location in the sequence. This mean‐field assumption reduces model complexity and describes the data well, as will be seen in the Results section. We account for the charges of the N‐ and C‐terminal amino and carboxyl groups, respectively, as well as the FRET dyes, by modifying the sequence appropriately; in one approach, dyes with charge ‐2 are incorporated by adopting the effective charge *q*_*m*_ = −2*α*_−_ (see Supporting Information Table S1 for specific sequences and Figure S1 and Section S3 for details of charge assignment for dyes and amino acids). The overall chain dimensions are given by a dimensionless swelling factor *x* defined as the ratio of the ensemble‐averaged mean squared end‐to‐end distance, $\left\langle R_{ee}^{2}\right\rangle$, and a Flory random coil reference state with mean squared end‐to‐end distance *Nb* *ℓ*, where *b* = 3.8 Å is the bond length, and ℓ = 8.0 Å is the Kuhn length (see References [13, 16] for details). The swelling factor $x=\langle R_{ee}^{2}\rangle /Nb\ell$ and the effective charge weights (degrees of ionization) *α*_+_, *α*_−_ depend on each other, so we require a self‐consistent description. Thus, we construct a free energy *F* as a function of (*x*, *α*_+_, *α*_−_) with five contributions:^[^ ^26^ ^]^ *F*(*x*, *α*_+_, *α*_−_) = *F*_1_(*α*_+_, *α*_−_) + *F*_2_(*α*_+_, *α*_−_) + *F*_3_(*α*_+_, *α*_−_) + *F*_4_(*α*_+_, *α*_−_) + *F*_5_(*x*, *α*_+_, *α*_−_). *F*_1_, *F*_2_ are the combinatorial and translational entropies of the ions bound to the chain and free in solution, respectively; *F*_3_ is the fluctuation contribution of all ions; *F*_4_ is the free energy (related to the equilibrium constant) of ion pair formation arising from each counter ion condensed with its oppositely charged partner on the chain, as in Ref. [26]; and *F*_5_ gives the free energy of the chain arising from chain connectivity and intra‐chain interactions. In Figure S2 (Supporting Information) and below we describe the details of these different free energy contributions.

#### 2.1.1 Details of Entropic and Fluctuation Contributions to the Free Energy

First, we describe the three contributions *F*_1_, *F*_2_ and *F*_3_. The free energy *F*_1_ corresponding to the combinatorial entropy of condensed counterions is given by

(1) $$\begin{array}{ccc} \frac{\beta F_{1}}{N} & = & f_{+}\left(\alpha_{+}\ln\left(\alpha_{+}\right)+\left(1-\alpha_{+}\right)\ln\left(1-\alpha_{+}\right)\right)\\ & & +\;f_{-}\left(\alpha_{-}\ln\left(\alpha_{-}\right)+\left(1-\alpha_{-}\right)\ln\left(1-\alpha_{-}\right)\right) \end{array}$$

where we introduce the charge fractions *f*_±_ = *N*_±_/*N*, and *β* = 1/*k*_*B*_ *T*. The free energy *F*_2_ corresponding to the translational entropy from counterions free in solution is given by

(2) $$\begin{array}{ccc} \frac{\beta F_{2}}{N} & = & \left(f_{+}\alpha_{+}+{\tilde{c}}_{s}/\tilde{\rho }\right)\ln\left(f_{+}\alpha_{+}\tilde{\rho }+{\tilde{c}}_{s}\right)+\left(f_{-}\alpha_{-}+{\tilde{c}}_{s}/\tilde{\rho }\right)\ln\left(f_{-}\alpha_{-}\tilde{\rho }+{\tilde{c}}_{s}\right)\\ & & -\;\left(f_{+}\alpha_{+}+f_{-}\alpha_{-}+2{\tilde{c}}_{s}/\tilde{\rho }\right) \end{array}$$

where $\tilde{\rho }$ and ${\tilde{c}}_{s}$ are the reduced concentrations of monomers and salt defined as $\tilde{\rho }\equiv \rho \;b^{3}$ and ${\tilde{c}}_{s}\equiv c_{s}\;b^{3}$, respectively. For example, given a concentration *ρ* in molar units (mol L^−1^) and length *b* in Angstroms (Å), the reduced equivalent is obtained via $\tilde{\rho }=\left(6.023\times 10^{-4}\;L\;{\mathrm{mol}}^{-1}/\;{Å}^{3}\right)\rho \;b^{3}$. We note that in our case, the contribution of the terms *f*_+_α_+_ and *f*_−_α_−_ is negligible for the counterion concentration, since the single‐molecule experiments were performed at picomolar IDP concentrations, but we include the terms in the equations for generality. The electrostatic free energy *F*_3_ from Debye–Hückel theory,^[^ ^43^ ^]^ also known as the ion fluctuation contribution,^[^ ^26^ ^]^ is

(3) $$\begin{array}{ccc} \frac{\beta F_{3}}{N} & = & -\frac{\kappa^{3}}{12\pi \rho }=-\frac{2\sqrt{\pi }}{3\tilde{\rho }}\;{\tilde{\ell }}_{B}^{\;3/2}{\left[\left(f_{+}\alpha_{+}+f_{-}\alpha_{-}\right)\tilde{\rho }+2{\tilde{c}}_{s}\right]}^{3/2} \end{array}$$

where

(4) $$\kappa^{2}=4\pi \ell_{B}\left[\left(f_{+}\alpha_{+}+f_{-}\alpha_{-}\right)\rho +2c_{s}\right]$$

with Bjerrum length *ℓ*_*B*_ = *e* ^2^/(4π*ϵ*_0_ *ϵ* *k*_*B*_ *T*), *ϵ*_0_ is the vacuum permittivity, and *ϵ* = 80 for water. We use the reduced Bjerrum length ${\tilde{\ell }}_{B}\equiv \ell_{B}/b$.

#### 2.1.2 Free Energy of Ion Pair Formation

Next, we consider the free energy of ion pair formation along the chain given by *F*_4_.^[^ ^26^, ^44^ ^]^ We write the energy of forming a single ion pair as an effective Coulomb interaction, *β*Δ*E* = −*ℓ*_*B*_ *δ*/*d*. One may interpret the ratio *δ*/*d* as a single sequence‐specific parameter; here, we keep the two parameters *δ* and *d* independent and emphasize the connection to the physical separation distance, which enables a consistent treatment for dipolar interactions (see ref. [28] for details). The effective separation distance, *d*, is defined by 1/*d* = [1/*p* + 1/(2*δ* *a*_1_) + 1/(2*a*_2_)]. Here, *p* is the physical separation distance, *a*_1_ is the radius of the small counterion, and *a*_2_ is the radius of the charged group of the side chain (see Chapter 5 of Ref. [45] for details). The charged side chain is much larger than the other length scales: *a*_2_ ≫ *a*_1_, *p*. We expect that the physical separation is similar to the size of a counterion: *p* ≈ *a*_1_. Thus, we take the approximate form 1/*d* ≈ (1/*p*) [1 + 1/(2*δ*)]. Upon counting the counterions condensed along the chain, and using the reduced dipole length $\tilde{p}=p/b$, and $\tilde{\ell_{B}}=\ell_{B}/b$, the total energy associated with ion pair formation is

(5) $$\frac{\beta F_{4}}{N}=-\left[f_{+}\left(1-\alpha_{+}\right)+f_{-}\left(1-\alpha_{-}\right)\right]\frac{{\tilde{\ell }}_{B}}{\tilde{p}}\left(\delta +\frac{1}{2}\right)$$

#### 2.1.3 Sequence‐Dependent Free Energy of the Polymer Chain

Finally, the detailed form of the chain free energy *F*_5_ is given by

(6) $$\begin{array}{ccc} \beta F_{5} & = & \frac{3}{2}\left(x-\ln\left(x\right)\right)+\frac{\omega_{3}B}{2}{\left(\frac{3}{2\pi x}\right)}^{3}\\ & & +\;2{\tilde{\ell }}_{B}Q{\left(\frac{3}{2\pi x}\right)}^{1/2}+\Omega {\left(\frac{3}{2\pi x}\right)}^{3/2} \end{array}$$

This free energy has four contributions: elastic entropy from chain connectivity (first term on the right hand side), three‐body repulsive excluded volume with strength *ω*_3_, two‐body long‐range electrostatics between two charges given by the *Q* term, and two‐body non‐electrostatic interactions (and other short‐range interactions such as charge‐dipole, dipole–dipole etc.) in Ω given by the last term. The three‐body contribution is given by

(7) $$B=\frac{1}{N}\sum_{l=3}^{N}\sum_{m=2}^{l-1}\sum_{n=1}^{m-1}\frac{\left(l-n\right)}{{\left[\left(l-m\right)\left(m-n\right)\right]}^{3/2}}$$

where *l*, *m*, *n* are the indices of residues participating in the three‐body interaction. The three‐body repulsive interaction is needed to prevent chain collapse in the case of strong intrachain attraction.^[^ ^46^, ^47^ ^]^ The sequence‐dependent charge‐charge electrostatic interactions with screening, described by *Q*, are defined as [19]

(8) $$\begin{array}{c} Q=\frac{1}{N}\sum_{m=2}^{N}\sum_{n=1}^{m-1}q_{m}q_{n}\;{\left(m-n\right)}^{1/2}\;A\left({\tilde{\kappa }}^{2}x\left(m-n\right)/6\right) \end{array}$$

(9) $$\begin{array}{c} A\left(z\right)=1-\sqrt{\pi z}\;\exp\left(z\right)\;\text{erfc}\left(\sqrt{z}\right) \end{array}$$

where the effective charge on each residue is dictated by the charge weights, *q*_*m*_ = ±*α*_±_. In the limit of zero screening and full ionization (*α*_±_ = 1), *Q* reduces to the Sequence Charge Decoration (SCD) metric defined in prior work.^[^ ^8^, ^16^ ^]^ The inverse Debye screening length is given in reduced form by ${\tilde{\kappa }}^{2}=4\pi {\tilde{\ell }}_{B}\left[\left(f_{+}\alpha_{+}+f_{-}\alpha_{-}\right)\tilde{\rho }+2{\tilde{c}}_{s}\right]$, where densities of residues and salt ions are, in reduced form, $\tilde{\rho }\equiv \rho b^{3}$ and ${\tilde{c}}_{s}\equiv c_{s}b^{3}$.

The two‐body interaction, given by Ω, has three distinct contributions. The first contribution is non‐electrostatic, Ω_non‐e_, given by

(10) $$\Omega_{\text{non-e}}=\frac{1}{N}\sum_{m=2}^{N}\sum_{n=1}^{m-1}\omega_{m,n}{\left(m-n\right)}^{-1/2}$$

where *ω*_*m*,*n*_ is the interaction between amino acids *m* and *n*. Since the interaction parameters between individual residues are unknown, we use an effective interaction parameter *ω*_2, *ee*_ (to compute the end‐to‐end distance) defined as

(11) $$\omega_{2,ee}=\left[\sum_{m=2}^{N}\sum_{n=1}^{m-1}\omega_{m,n}{\left(m-n\right)}^{-1/2}\right]/\left[\sum_{m=2}^{N}\sum_{n=1}^{m-1}{\left(m-n\right)}^{-1/2}\right]$$

Thus, *ω*_2,*ee*_ is a pairwise mean‐field non‐electrostatic interaction among all residues. Sequence specificity of *ω*_2,*ee*_ has been termed Sequence Hydropathy Decoration (SHD) and was used to model simulated chain dimensions.^[^ ^13^ ^]^ More recently, it has been determined by a machine learning model using a large set of coarse‐grained simulations.^[^ ^48^ ^]^ With this definition, we get

(12) $$\Omega_{\text{non-e}}=\omega_{2,ee}\frac{1}{N}\sum_{m=2}^{N}\sum_{n=1}^{m-1}{\left(m-n\right)}^{-1/2}$$

Two additional contributions to Ω are Ω_c‐d_ and Ω_d‐d_, resulting from directionally averaged charge–dipole and dipole–dipole interactions approximated as delta function potentials,^[^ ^26^, ^45^ ^]^ with

(13) $$\begin{array}{ccc} \Omega_{\text{c-d}} & = & \omega_{cd}\frac{1}{N}\sum_{m=2}^{N}\sum_{n=1}^{m-1}\left(c_{m}d_{n}+c_{n}d_{m}\right){\left(m-n\right)}^{-1/2} \end{array}$$

(14) $$\begin{array}{ccc} \Omega_{\text{d-d}} & = & \omega_{dd}\frac{1}{N}\sum_{m=2}^{N}\sum_{n=1}^{m-1}d_{m}d_{n}\;{\left(m-n\right)}^{-1/2} \end{array}$$

where, for each residue, the charge state is determined by the unsigned weights (probabilities of ionization), *c*_*m*_ = *α*_±_, and the dipole state is dictated by the complementary weights (probabilities of condensation), *d*_*m*_ = 1 − *α*_±_. Non‐ionizable residues do not contribute. The specific arrangement of charges in the sequence will make these contributions sequence‐specific. Consequently, we get two new patterning metrics: Sequence Charge‐Dipole Decoration (*SCDD* = Ω_c‐d_/*ω*_*cd*_) and Sequence Dipole Decoration (*SDD* = Ω_d‐d_/*ω*_*dd*_) as defined in ref. [28]. The magnitudes of the effective interaction (pseudopotential) arising from charge–dipole (*ω*_*cd*_) and dipole–dipole (*ω*_*dd*_) contributions are given by [26, 44, 45],

(15) $$\begin{array}{ccc} \omega_{cd} & = & -\frac{\pi }{3}\delta^{2}{\tilde{\ell }}_{B}^{2}{\tilde{p}}^{2}\exp\left(-2\tilde{\kappa }\right)\left[2+\tilde{\kappa }\right] \end{array}$$

(16) $$\begin{array}{ccc} \omega_{dd} & = & -\frac{\pi }{9}\delta^{2}{\tilde{\ell }}_{B}^{2}{\tilde{p}}^{4}\exp\left(-2\tilde{\kappa }\right)\left[4+8\tilde{\kappa }+4{\tilde{\kappa }}^{2}+{\tilde{\kappa }}^{3}\right] \end{array}$$

Thus, the salt concentration dependence of the chain dimensions is not only determined by screening of charge–charge interactions (given by Equation 8) but also by these dipole‐induced patterning metrics.

#### 2.1.4 Free Energy Minimization Scheme

The free energy thus depends on three adjustable parameters (Figure S2, Supporting Information): *ω*_2,*ee*_ accounting for sequence‐specific non‐electrostatic interactions that we cannot predict from first principles at present; *δ*, the dielectric mismatch between the bulk solvent and at a position in the vicinity of the chain; and *p*, the dipole length describing the distance between the charged group of the side chain and the neutralizing ion. All other parameters are known. By minimizing this free energy for given values of *ω*_2,*ee*_, *p*, and *δ* with respect to *x*, *α*_+_, *α*_−_, we self‐consistently determine the optimal effective charge and ensemble‐averaged end‐to‐end distance to compare against experimentally measured dye‐dye distances. We determine *ω*_2,*ee*_ from the FRET measurements at high salt (0.4 M), where charge interactions are screened, and we use *p* and/or *δ* as adjustable parameters in the fit to the experimental data. We used this formalism recently for describing the salt‐dependent end‐to‐end measurements of ProTα and its net charge.^[^ ^28^ ^]^

#### 2.1.5 Different Model Variants Used

We use various forms of this model to identify the one that provides the best balance between describing the experimental FRET data and the simplicity of the model, especially the number of adjustable parameters. The first model is the simple case in which counter ion condensation is excluded; this case is recovered from the formulation above by setting *α*_±_ = 1, and it does not require parameters *p* and *δ*. We call this the *full ionization model* and use the label M0. A related model is a mean‐field polyampholyte model developed by Higgs and Joanny^[^ ^49^ ^]^ (here referred to as “H‐J”) that does not adjust charge weights and is not sensitive to sequence charge patterning; we amend the original formulation with a three‐body term as in the second term of Equation (6), with Equation (7), to prevent unphysical chain collapse (see Section S5 and Equations S1– S3, Supporting Information, for the details of the H‐J model).

For the cases with counter ion condensation, the models differ by the method for determining *p* and *δ*. We find that a single fixed value of the dipole length, *p*/*b* = 0.66, is suitable for all sequences. However, *δ* is expected to depend on the local dielectric permittivity near the chain, which can depend on charge patterning and chain dimensions, and is therefore sequence‐dependent. We thus define M1 as the case where *δ* is determined individually from chi‐square fitting of the FRET‐basefd end‐to‐end distances for each of the 16 linker IDRs previously selected for diversity in amino acid composition, hydrophobicity, and charge patterning^[^ ^36^ ^]^ (Table S1, Supporting Information) as a function of salt concentration (while *ω*_2,*ee*_ was adjusted to match the high‐salt data point). This procedure yields a linear trend relating *δ* to low‐salt end‐to‐end distance (see Results). In a third model, M2, *δ* is determined from the low‐salt end‐to‐end distances based on this linear trend (again adjusting *ω*_2,*ee*_), thus substantially reducing the number of fit parameters.

We do not explicitly model charge regulation, i.e., changes in the protonation state of the amino acids, since all experiments were performed near pH 7 and at salt concentrations above 50 mM. Under these conditions, the protonation states can be approximated based on the nominal *pK*_*a*_ values of the ionizable groups.^[^ ^27^, ^50^ ^]^ Thus, we neglect deviations that might occur at very low salt concentrations below the range we probe, and for histidine, which is present only in very low proportions in the sequences we investigate. If charge reduction occurs due to both charge regulation and charge renormalization, our formalism, when fitted to the data, will yield a single set of mean‐field parameter values that effectively account for both effects. More advanced variants of the theory that separate protonation effects and the complexation between charged residues and salt ions^[^ ^51^ ^]^ could be developed based on suitable pH‐dependent experimental data.

### 2.2 Model For Computing Intrachain Distance Maps

Based on the single‐molecule FRET experiments, we have not only measured average end‐to‐end distances but also distances $\left\langle R_{ij}^{2}\right\rangle$ between residues at positions *i*, *j*. Such experiments were performed for two IDPs, Stm^[^ ^37^ ^]^ and ProTα,^[^ ^3^, ^38^ ^]^ which are much longer than the linker IDRs (see Table S1, Supporting Information, for details of the sequences and dye positions). To determine $\left\langle R_{ij}^{2}\right\rangle$ in the context of our theory, we define residue pair (*i*, *j*)‐specific swelling factors *x*_*i*, *j*_ just like *x* defined above, and construct a free‐energy function *F* that depends on *x*_*ij*_, *α*_+_, *α*_−_. For this approach, *F*_1_, *F*_2_, *F*_3_, and *F*_4_ are defined as above, only the chain free energy *F*_5_ is now constructed in the reaction coordinate *x*_*ij*_ as:

(17) $$\begin{array}{ccc} \beta F_{5} & = & \frac{3}{2}\left(x_{ij}-\ln\left(x_{ij}\right)\right)+\frac{\omega_{3}T_{ij}}{2\;|i-j|}{\left(\frac{3}{2\pi x_{ij}}\right)}^{3}\\ & & +\;2{\tilde{\ell }}_{B}Q_{ij}{\left(\frac{3}{2\pi x_{ij}}\right)}^{1/2}+\Omega_{ij}{\left(\frac{3}{2\pi x_{ij}}\right)}^{3/2} \end{array}$$

where *T*_*ij*_ is the residue pair‐specific three‐body term defined in our earlier work,^[^ ^16^ ^]^ and *Q*_*ij*_ is the patterning metric arising from charge–charge interactions specific to residue pair *i*, *j*, also known as the Sequence Charge Decoration Matrix (SCDM),^[^ ^16^ ^]^ and defined as

(18) $$\begin{array}{ccc} Q_{i,j} & = & \frac{1}{\left(i-j\right)}\left[\sum_{m=j}^{i}\sum_{n=1}^{j-1}q_{m}q_{n}\frac{{\left(m-j\right)}^{2}}{{\left(m-n\right)}^{3/2}}A\left(\kappa^{2}b^{2}\left(m-n\right)x_{i,j}/6\right)\right.\\ & & +\;\sum_{m=j+1}^{i}\sum_{n=j}^{m-1}q_{m}q_{n}{\left(m-n\right)}^{1/2}A\left(\kappa^{2}b^{2}\left(m-n\right)x_{i,j}/6\right)\\ & & +\;\sum_{m=i+1}^{N}\sum_{n=1}^{j-1}q_{m}q_{n}\frac{{\left(i-j\right)}^{2}}{{\left(m-n\right)}^{3/2}}A\left(\kappa^{2}b^{2}\left(m-n\right)x_{i,j}/6\right)\\ & & +\;\left.\sum_{m=i+1}^{N}\sum_{n=j}^{i}q_{m}q_{n}\frac{{\left(i-n\right)}^{2}}{{\left(m-n\right)}^{3/2}}A\left(\kappa^{2}b^{2}\left(m-n\right)x_{i,j}/6\right)\right] \end{array}$$

with *A* defined in Equation (9) above.

The two‐body interaction Ω_*ij*_ is also residue pair‐specific and accounts for three contributions: 1) purely non‐electrostatic interactions (also defined as Sequence Hydropathy Decoration Matrix or *SHDM* ^[^ ^16^, ^52^ ^]^); 2) sequence‐dependent charge–dipole interactions (Sequence Charge–Dipole Decoration Matrix, *SCDDM*); and 3) sequence dipole‐dipole interactions (sequence Dipole Decoration Matrix, *SDDM*). These patterning contributions are now dependent on the specific residue pair (*i*, *j*) and hence give rise to matrices generalizing the metrics defined for the end‐to‐end distance. In our previous work,^[^ ^16^ ^]^ the *SHDM* was derived as

(19) $$\begin{array}{ccc} & & SHDM_{i,j}\\ & & \;=\frac{1}{\left(i-j\right)}\left[\sum_{m=j}^{i}\sum_{n=1}^{j-1}\omega_{m,n}\frac{{\left(m-j\right)}^{2}}{{\left(m-n\right)}^{5/2}}+\sum_{m=j+1}^{i}\sum_{n=j}^{m-1}\omega_{m,n}{\left(m-n\right)}^{-1/2}\right.\\ & & \;\left.+\sum_{m=i+1}^{N}\sum_{n=j}^{i}\omega_{m,n}\frac{{\left(i-n\right)}^{2}}{{\left(m-n\right)}^{5/2}}+\sum_{m=i+1}^{N}\sum_{n=1}^{j-1}\omega_{m,n}\frac{{\left(i-j\right)}^{2}}{{\left(m-n\right)}^{5/2}}\right]. \end{array}$$

As noted above, the *ω*_*m*,*n*_ values are not known a priori. However, as previously done, the entire contribution is written as a single parameter *ω*_2,*i*, *j*_, where *SHDM*_*i*,*j*_ is normalized by the interaction‐independent segmental part $N_{i,j}$ (i.e., setting *ω*_*m*,*n*_ = 1 in Equation 19). Specifically, we have

(20) $$\begin{array}{c} \omega_{2,i,j}=SHDM_{i,j}/N_{i,j}. \end{array}$$

and

(21) $$\begin{array}{ccc} N_{i,j} & = & \frac{1}{\left(i-j\right)}\left[\sum_{m=j}^{i}\sum_{n=1}^{j-1}\frac{{\left(m-j\right)}^{2}}{{\left(m-n\right)}^{5/2}}+\sum_{m=j+1}^{i}\sum_{n=j}^{m-1}{\left(m-n\right)}^{-1/2}\right.\\ & & \left.+\sum_{m=i+1}^{N}\sum_{n=j}^{i}\frac{{\left(i-n\right)}^{2}}{{\left(m-n\right)}^{5/2}}+\sum_{m=i+1}^{N}\sum_{n=1}^{j-1}\frac{{\left(i-j\right)}^{2}}{{\left(m-n\right)}^{5/2}}\right] \end{array}$$

This definition is a generalization of *ω*_2,*ee*_ defined above (Equation 11) for the calculation of the end‐to‐end distance. In the limit of a homopolymer, where *ω*_*m*,*n*_ variation is zero, *ω*_2,*ee*_ = *ω*_2,*i*,*j*_ will be a constant independent of *i* and *j*. Otherwise, *ω*_2,*i*,*j*_ will depend on *i*, *j* and will be sequence‐specific. We determine *ω*_2,*i*, *j*_ by using the experimentally observed residue pair‐specific chain dimensions $\left\langle R_{ij}^{2}\right\rangle$ in the high‐salt limit to determine the non‐electrostatic interaction strength.

The sequence‐dependent charge–dipole interaction written in matrix form (due to specific residue pairs), *SCDDM*, is defined as

(22) $$\begin{array}{ccc} SCDDM_{i,j} & = & \omega_{cd}\frac{1}{\left(i-j\right)}\left[\sum_{m=j}^{i}\sum_{n=1}^{j-1}\left(c_{m}d_{n}+c_{n}d_{m}\right)\frac{{\left(m-j\right)}^{2}}{{\left(m-n\right)}^{5/2}}\right.\\ & & +\;\sum_{m=j+1}^{i}\sum_{n=j}^{m-1}\left(c_{m}d_{n}+c_{n}d_{m}\right){\left(m-n\right)}^{-1/2}\\ & & +\;\sum_{m=i+1}^{N}\sum_{n=j}^{i}\left(c_{m}d_{n}+c_{n}d_{m}\right)\frac{{\left(i-n\right)}^{2}}{{\left(m-n\right)}^{5/2}}\\ & & \left.+\sum_{m=i+1}^{N}\sum_{n=1}^{j-1}\left(c_{m}d_{n}+c_{n}d_{m}\right)\frac{{\left(i-j\right)}^{2}}{{\left(m-n\right)}^{5/2}}\right]. \end{array}$$

*SDDM* is defined as

(23) $$\begin{array}{ccc} SDDM_{i,j} & = & \omega_{dd}\frac{1}{\left(i-j\right)}\left[\sum_{m=j}^{i}\sum_{n=1}^{j-1}d_{m}d_{n}\frac{{\left(m-j\right)}^{2}}{{\left(m-n\right)}^{5/2}}\right.\\ & & +\sum_{m=j+1}^{i}\sum_{n=j}^{m-1}d_{m}d_{n}{\left(m-n\right)}^{-1/2}\\ & & +\sum_{m=i+1}^{N}\sum_{n=j}^{i}d_{m}d_{n}\frac{{\left(i-n\right)}^{2}}{{\left(m-n\right)}^{5/2}}\\ & & \left.+\sum_{m=i+1}^{N}\sum_{n=1}^{j-1}d_{m}d_{n}\frac{{\left(i-j\right)}^{2}}{{\left(m-n\right)}^{5/2}}\right], \end{array}$$

where ω_*cd*_, *ω*_*dd*_, *c*_*m*_, *d*_*m*_ have the same meaning as defined above.

As before, this function is minimized to determine optimal swelling factors *x*_*ij*_ and degrees of ionization *α*_±_ for a given residue pair *i*, *j*. The swelling factors are related to distances by $x_{ij}=\left\langle R_{ij}^{2}\right\rangle /(|i-j|b\ell )$. We fitted *ω*_2,*i*,*j*_ specific to residue pairs based on the experimental data with FRET labels in different positions, while *p* and *δ* were assumed to be independent of the segment probed and kept invariant for a given protein. We used experimentally measured *C*_α_ − *C*_α_ distances for these comparisons, and dyes were modeled as one residue with an effective charge of −2 (for Alexa 488 and 594), −1 (for CF6606R), or 0 (for Cy3B), without internal structure, in contrast to the analysis for the linker IDRs (see Section S3, Supporting Information). This formalism was also used to predict normalized distance maps $d_{i,j}^{*}$ (see Section S6, Supporting Information) for a given linker sequence with the assumption *ω*_2,*ee*_ = *ω*_2,*i*,*j*_, and *ω*_2,*ee*_ determined by fitting the single‐molecule FRET data for the end‐to‐end distances of the 16 sequence‐diversified linker IDRs.^[^ ^36^ ^]^

## 3 Results

We performed single‐molecule FRET experiments on 18 different IDPs and over a wide range of KCl concentrations to provide a data set that allows us to stringently test models that describe intrachain distances and their salt‐dependence. By placing FRET donor and acceptor dyes at different positions in the sequence, we can probe not only end‐to‐end distances but also shorter segments within the IDPs. To cover a broad range of sequences, we use a combination of samples (Figure 1). With a focus on sequence composition, we measured the end‐to‐end distances in a recently established set of 16 sequences selected from intrinsically disordered regions (IDRs) connecting RNA‐binding domains of identical length of 57 residues, but with large differences in amino acid composition, hydrophobicity, and charge patterning^[^ ^36^ ^]^ (Table S1, Supporting Information). While the previous study^[^ ^36^ ^]^ reported measurements only at a single salt concentration, here we performed measurements across different salt concentrations to rigorously test different electrostatic models. To complement these measurements and test the results also for segments within longer IDPs, we use Starmaker (Stm)^[^ ^37^ ^]^ and Prothymosin α (ProTα),^[^ ^3^, ^38^ ^]^ two naturally occurring, highly negatively charged IDPs of 598 and 112–113 residues, respectively (Table S1, Supporting Information). We note that naturally evolved sequences have the advantage of higher success rates in recombinant expression for protein production and avoid issues such as translation arrest of long polylysine repeats.^[^ ^53^ ^]^ Moreover, contributions other than charge interactions are important for a realistic parametrization of the theory, so the content of uncharged amino acid residues is an essential aspect of our sequence set. To further account for the contribution of the dyes, especially their charges,^[^ ^3^, ^36^ ^]^ we labeled the 16 IDRs with two different FRET pairs, on the one hand AlexaFluor 488 and 594, and on the other Cy3B and CF660R. The Alexa dyes carry a net charge of –2 each, whereas Cy3B is a net neutral zwitter ion, and CF660R carries a net charge of –1 (see Section S3, Supporting Information). For all of these different sequences and variants, we performed measurements across a broad range of salt concentrations to modulate the electrostatic interactions.

Based on the FRET data, we use the following strategy: We infer average inter‐dye distances for the 16 IDRs from transfer efficiencies measured as a function of monovalent salt concentration using the SAW‐ν distance distribution (see Section S2, Supporting Information),^[^ ^54^, ^55^ ^]^ which has previously been shown to yield monomodal distance distributions for the 16 IDRs that agree well with those from coarse‐grained and atomistic implicit‐solvent simulations;^[^ ^36^ ^]^ we then use the resulting average distances for direct comparison with the values from the different theoretical models described above, where the charge interactions are assumed to be sufficiently screened at the highest salt concentrations used (>0.4 M) so that those distances can be used for estimating the non‐electrostatic contribution to the free energy of the chains. By minimizing the deviation between experiment and theory for all 16 sequences and all salt concentrations globally, we obtain parameter sets that provide the best description of all sequences for each model. It is worth emphasizing that the sequences even exhibit qualitatively different trends with increasing salt concentration: some sequences compact, while others expand, reflecting the importance of the content and distribution of positive and negative charges within the sequence. This rich set of data collected for these diverse linker IDRs and two additional IDPs Stm and ProTα provides the first rigorous test of a unified analytical theory of electrostatics that self‐consistently accounts for the effects of sequence, variable charge states, dipolar interactions, ionic strength and their impact on the chain conformation.

### 3.1 Full Ionization is Insufficient for Describing Chain Dimensions Quantitatively

**Figure 2:**
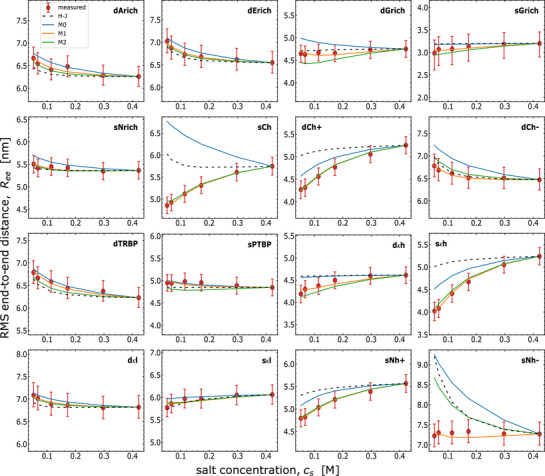
Comparison of the different theoretical models with single‐molecule FRET results for 16 sequence‐diversified IDRs labeled with Alexa 488/594 (“measured”). Full ionization models, purely compositional (Higgs‐Joanny; H‐J, black dashed line), or with sequence dependence (M0, blue line), tend to overestimate conformations compared to FRET‐based RMS end‐to‐end distances across ionic strengths. Counterion models, M1 (orange line) with sequence‐dependent δ, and the simpler near‐predictive M2 (green line) with sequence‐dependent δ given by the linear relation $\delta =-0.123\;\sqrt{\left\langle R_{ee}^{2}\right\rangle }+2.146$ from low‐salt $\sqrt{\left\langle R_{ee}^{2}\right\rangle }$ (in nm) (see Figure 3), both with $\tilde{p}=0.66$, describe the data better (see Table 1). For all models, the two‐body non‐electrostatic interaction parameter ω_2, *ee*_ is determined by matching the data points at the highest salt concentration. Models M0, M1, M2 used a three‐body non‐electrostatic interaction strength of ω_3_ = 0.1. Higgs–Joanny (H–J) additionally required a strongly amplified three‐body term, ω_3_ = 10, to prevent unphysical collapse of polyampholytic sequences at low salt. See Table S2 (Supporting Information) for parameter values.

We begin with the simplest model, M0, to test its ability to describe the data. Model M0 only minimizes *x* and assumes *α*_±_ = 1, implying that all charged residues are fully ionized. While M0 correctly captures the qualitative trends of salt‐induced compaction/expansion of the majority of the sequences, it tends to overestimate the chain dimension in the low‐salt regime (**Figure** **2**). For the sequence sCh, M0 even fails to describe the qualitative trend, predicting a decrease in chain dimensions with increasing salt concentration, in contradiction to the data. For comparison, we also include the mean‐field polyampholyte model of Higgs and Joanny,^[^ ^3^, ^49^ ^]^ which assumes the positive and negative charges to be distributed uniformly along the sequence and does therefore not take charge patterning into account. The resulting trends and quantitative agreement are generally similar to those of model M0. However, for a substantial fraction of the sequences, neither of these simple models provide a quantitative account of the experimental findings.

### 3.2 Model With Ion Condensation Describes Data Well Across IDR Sequences

To explain the ionic‐strength‐dependent data of all 16 linker IDR sequences, we thus test the model with ion condensation. An important aspect of model selection is to avoid overfitting. For this purpose, we apply a model with the minimal number of parameters. Although all three adjustable parameters are expected to be sequence‐dependent, we expect the average dipole length *p* to be least sensitive to the sequence because it is not explicitly dependent on the local chain environment, while *δ* is dependent on local permittivity. Thus, we fit the data with model M1 where *p* is held fixed but *δ* is allowed to vary from sequence to sequence (see Section S8, Supporting Information, for details on our χ^2^ fitting procedure).) We also determine the non‐electrostatic parameter *ω*_2,*ee*_ for each sequence by matching the high‐salt data, where electrostatics is expected to be largely screened.^[^ ^3^ ^]^ The best fit value of the dimensionless dipole length $\tilde{p}=0.66$ and the values of *δ* (reported in Table S2, Supporting Information) for each sequence are within the expected range (see Section S7, Supporting Information). Model M1 fits the salt‐dependent chain dimensions very well for virtually all linker sequences (Figure 2). We also note that the effective charge reduction due to counterion condensation can be pronounced (Figure S3, Supporting Information). Furthermore, the effective charges can differ substantially from sequence to sequence, and although they always decrease with increasing salt concentration, the amplitude of the change differs between sequences and between anionic and cationic groups.

**Figure 3:**
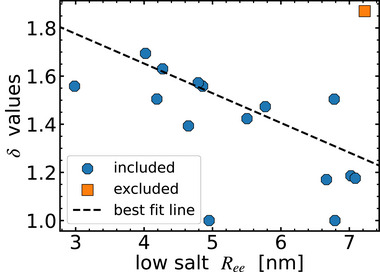
The dielectric mismatch parameter *δ* of all linker IDRs from model M1 (points) labeled with Alexa 488/594 is linearly correlated with the FRET‐based distances $\sqrt{\left\langle R_{ee}^{2}\right\rangle }$ (at 52 mM salt concentration), with a Pearson correlation coefficient of *r* = −0.64. Assuming a linear dependence of *δ* on $\sqrt{\left\langle R_{ee}^{2}\right\rangle }$ at low salt (52 mM), we find the optimized dependence (black dashed line) by fitting $\left\langle R_{ee}^{2}\right\rangle$ as a function of salt concentration (from FRET) for all 15 sequences simultaneously (all except the outlier sNh‐, orange). For model M2, *δ* is set for each sequence according to this optimal line (black dashed line) given by $\delta =-0.123\;\sqrt{\left\langle R_{ee}^{2}\right\rangle }+2.146$, thus removing all parameters adjustable individually for each chain except the non‐electrostatic interaction strength *ω*_2,*ee*_.

The above analysis with M1 yields fitted *δ* values with a substantial spread from roughly 1.0–1.9 (Table S2, Supporting Information), precluding the use of a global value. Understanding the sequence‐dependent variation of *δ* is important to reduce model complexity and build a predictive model. We hypothesize that sequences with more compact dimensions would tend to have a greater mismatch between bulk and local dielectric (larger *δ*) due to lower solvent accessibility. To test this hypothesis, we plot *δ* against $\sqrt{\left\langle R_{ee}^{2}\right\rangle }$ at low salt concentration (**Figure** **3**) and notice a modest anticorrelation, with sNh‐ and sPTBP being two outliers. The additional scatter indicates contributions of other local effects (e.g., polar residues, spatial variation of dielectric mismatch) on *δ* beyond just the overall chain compactness. Nevertheless, to reduce the number of adjustable parameters, we leverage the linear correlation to find the simplest relation that describes the salt concentration‐dependent data for all sequences with a global fit. In this global fitting procedure, *ω*_2,*ee*_ is determined for each sequence by matching the high‐salt $\left\langle R_{ee}^{2}\right\rangle$ measurement. The linear relation between *δ* and $\sqrt{\left\langle R_{ee}^{2}\right\rangle }$ at low salt is adjusted until the total χ^2^ for 15 IDRs (all except sNh‐) is minimized. Note that this is different from directly fitting sequence‐specific *δ* values (blue points in Figure 3) obtained from M1 by fitting the salt concentration‐dependent data for each sequence separately. The sequence‐dependent value of *δ* obtained from the global fit can be estimated from the low‐salt (52 mM) mean squared end‐to‐end distance $\sqrt{\left\langle R_{ee}^{2}\right\rangle }$ (in nanometers) from the equation

(24) $$\delta =-0.123\;\sqrt{\left\langle R_{ee}^{2}\right\rangle }+2.146$$

where the minimum allowed value of *δ* is unity since the dielectric in the volume occupied by the chain cannot be higher than that of water. We find that the resulting model M2, in which *δ* values are used based on this optimized line (dashed black line in Figure 3), describes the data well for most sequences (Figure 2), including sPTBP, which was an outlier in Figure 3, implying that sPTBP is not very sensitive to the dielectric mismatch parameter.

**Figure 4:**
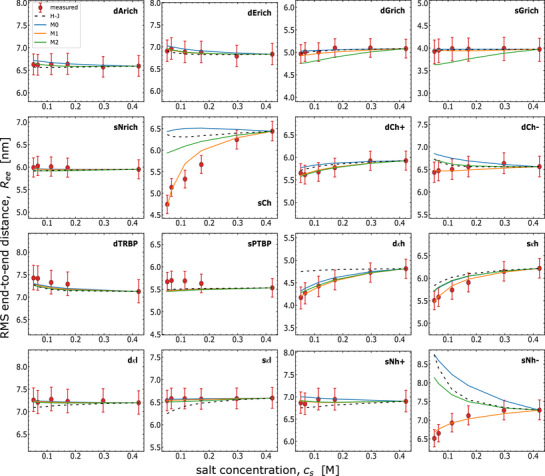
Comparison of the different theoretical models with single‐molecule FRET results for the 16 sequence‐diversified IDRs labeled with Cy3B/CF660R (“measured”) (analogous to Figure 2): compositional Higgs–Joanny (H–J, black dashes), sequence‐dependent model with full ionization (M0, blue line), counterion model with fitting *δ* individually (M1, orange line) and applying the near‐predictive linear relation for *δ* with $\sqrt{\left\langle R_{ee}^{2}\right\rangle }$ (M2, green line), all with $\tilde{p}=0.66$. The near‐predictive model M2 presented here is based on the same linear relation for predicting *δ* based upon the Alexa dye results shown in Figure 3), and still compares reasonably well with the FRET‐based RMS end‐to‐end distances across ionic strengths. Parameter values are shown in Table S3 (Supporting Information).

The only sequence that M2 fails to describe is sNh‐, which is also an outlier in the dependence of *δ* on $\sqrt{\left\langle R_{ee}^{2}\right\rangle }$ and was thus not included in the linear fit. sNh‐ is high in negative charge, with the highest net charge per residue among the IDRs of −0.35, and low in positive charge. Polyelectrolyte theory would thus predict a decrease in chain dimensions with the addition of salt, although there are contiguous stretches of 12 glutamates at the C‐terminus and 3 lysines at the N‐terminus, whose attractive interaction could lead to polyampholyte‐like expansion with increasing salt concentration.^[^ ^21^, ^49^ ^]^ However, the single‐molecule FRET measurements show that the dimensions of sNh‐ are largely insensitive to salt concentration. Within our model, this trend could be explained by a low degree of ionization or equivalently a high propensity to form dipoles by counterion condensation. A high value of dielectric mismatch promotes such dipole formation and a resulting low degree of ionization. The specific cause of such high dielectric mismatch is, however, unclear. On the other hand, it is possible that sNh‐ exhibits salt insensitivity due to other factors not included in the model. For example, it is conceivable that the dye interacts with the three lysine residues close to the N‐terminus and forms local contacts not accounted for in the model.^[^ ^36^ ^]^ Indeed, an influence of dye interactions is supported by the difference between the salt concentration dependence observed for the Alexa 488/594‐ and Cy3B/CF660R‐labeled variants (**Figure** **4**). There might thus be a competition between interactions of the lysine‐rich region with the glutamate‐rich region on the one hand and the N‐terminal dye on the other; the latter is expected to be less pronounced for Cy3B and CF660R owing to their lower net charge compared to the Alexa dyes.^[^ ^36^ ^]^ The salt insensitivity could also arise from specific non‐electrostatic interactions that are only described at a mean‐field level in our model.

Nevertheless, model M2 accounting for partial charge neutralization describes the data fairly well for all proteins except sNh‐. **Table** **1** provides a comparison between the four models (H–J, M0, M1, and M2). The models M1 and M2 taking into account counterion condensation perform better than the models without condensation (HJ and M0). The near‐predictive model M2 does only slightly worse than model M1 with *δ* adjusted individually for each sequence. Thus, model M2 reduces the number of adjustable parameters without significantly compromising its ability to describe the data, and thus increases the predictive power of our formalism. To further facilitate the comparison of the models, we present the deviations of all four models from the experimental data in Figure S4 (Supporting Information). Positive differences ($\Delta R_{ee}\equiv \Delta \sqrt{\left\langle R_{ee}^{2}\right\rangle }$) are where theory predicts more expanded conformations than measured, while negative differences are where theory predicts more compact conformations. We also provide comparison of *χ* ^2^ errors across models for each sequence (Figure S5, Supporting Information).

**Table 1:** Summary of model performance for the 16 IDRs with Alexa dyes for Higgs–Joanny (H–J), full ionization (model M0), counterion model with fitted *δ* (model M1), counterion model with *δ* from best linear dependence on low‐salt chain dimensions $\sqrt{\left\langle R_{ee}^{2}\right\rangle }$ (model M2). Mean errors (chi‐squared) of $\sqrt{\left\langle R_{ee}^{2}\right\rangle }$ across salt concentrations are determined for each sequence, then we report two means: i) the mean across all sequences (overall mean) and ii) the mean across all except sNh‐ (excluding sNh‐). A comparison of *χ* ^2^ across models for each sequence is shown in Figure S5 (Supporting Information).

	H–J	M0	M1	M2
Overall mean	3.297	4.702	0.0397	0.700
excluding sNh‐	2.586	3.725	0.0348	0.166

The inferred parameter values can be further used to quantitatively determine the different contributions to the total free energy (entropy of counterions, free energy of ion condensation, chain free energy, etc). For this purpose, we selected two representative sequences, sκh and dCh‐ due to their differences in charge composition and salt response. sκh is a strong polyampholyte with 11 positive and 11 negative charges, and expands with addition of salt. On the other hand, dCh‐ is polyelectrolyte‐like, with 13 negative charges and 4 positive charges and compacts with addition of salt. Figure S6 (Supporting Information) shows the contributions of the different free energy terms for these two sequences using the parameter values obtained from model M1.

As detailed above, model M2 reduces the number of parameters by estimating *δ* from the measured end‐to‐end distance at low salt and ignores its variation with salt. This is a reasonable approximation given the greater variation in the end‐to‐end distance at low salt across different sequences than as a function of salt concentration for a given sequence. However, the formalism can be extended to model M3, where δ is allowed to vary with salt for a given sequence. The salt dependence of *δ* can be approximated using equation 24, where $\sqrt{\left\langle R_{ee}^{2}\right\rangle }$ is the end‐to‐end distance measured at different salt concentrations. Model M3 compares well with the data (Figure S7, Supporting Information). We use this analysis primarily as a consistency check, since it is difficult to use M3 for predictions if salt‐dependent measurements are not available. Variation in *δ* can also arise from the decrease in dielectric constant with increasing salt concentration, known as dielectric decrement.^[^ ^56^ ^]^ However, for KCl and the highest salt concentration of 420 mM used here, the change in dielectric will be relatively small (from 80 to 75) with minimal impact on *δ* values. Thus, we conclude that, overall, M2 is the preferred model for its simplicity, practical use, and its ability to describe a wide range of data.

### 3.3 Testing the Model With a Different Dye Pair

To address the question of how much the charged dyes impact chain dimensions and whether our models are general enough to describe the effect, we collected ionic strength‐dependent single‐molecule FRET data using a different pair of dyes, Cy3B and CF660R, which carry a lower net charge than the AlexaFlours and have been found to exhibit less attractive interactions with positively charged residues.^[^ ^36^ ^]^ We then used models M0, M1, and M2 to fit the data, with the same value of *p* and the same linear relation between *δ* and $\sqrt{\left\langle R_{ee}^{2}\right\rangle }$ used for the Alexa dyes (Figure 3 and Equation 24) to estimate the dielectric mismatch for M2. The only independently adjustable parameter is the non‐electrostatic interaction *ω*_2,*ee*_ determined from the high‐salt data for each sequence. The resulting model parameters are listed in Table S3 (Supporting Information). As before, we find that both M1 and M2 describe the data well, with the exception of sNh‐ and sCh, for which M2 does not provide a good fit (Figure 4). Both sNh‐ and sCh show a strong salt‐induced expansion in the experimental data, indicative of a pronounced polyampholyte effect. Indeed, sNh‐ and sCh are the two sequences with the combination of the highest total charge content and highest degree of charge segregation in the set.^[^ ^36^ ^]^ The accumulation of opposite net charges near the termini may thus have a pronounced influence on chain dimensions, potentially modulated by charge‐mediated dye interactions. We note that our model ignores the branched nature of the dye moieties attached via flexible linkers and any salt dependence of chain dimensions arising from mechanisms other than electrostatic effects. For example, the solubility of polar amino acids (and fluorophores) can vary with salt concentration, potentially modulating the salt‐dependent variation of the chain dimensions.^[^ ^57^ ^]^ We also ignored the spatial variation in *δ* for a given sequence even at a fixed salt concentration. However, differences in local compactness and the nature of chemical groups may cause fluctuations in the *δ* values. It is important to note that the data for sCh can be well described by M1, which optimizes the dielectric mismatch parameter within a range for each sequence individually, yielding a value of *δ* = 2.03, whereas for M2 the value is *δ* = 1.56.

As before, we compare all models (H–J, M0, M1, M2) for the 16 IDRs with Cy3B/CF660R in **Table** **2** and Figure 4. Overall, we find that M1 is the best model in terms of absolute agreement with the data, but at the price of individually adjusted values of *δ*. The near‐predictive model M2 performs better than both the full ionization (M0) and H‐J model, and given the fixed relation between *δ* and $\sqrt{\left\langle R_{ee}^{2}\right\rangle }$, it provides a good compromise between fit quality and the number of adjustable parameters.

**Table 2:** Summary of model performance for the 16 IDRs with Cy3B/CF660R dyes using Higgs–Joanny (H–J), full ionization model (M0), counterion model with individually fitted *δ* (model M1), counterion model with *δ* from best linear dependence on low‐salt $\sqrt{\left\langle R_{ee}^{2}\right\rangle }$ (model M2). Mean errors (chi‐square) of $\sqrt{\left\langle R_{ee}^{2}\right\rangle }$ across salt concentrations are determined for each sequence, then we report two means: i) the mean across all sequences (overall mean) and ii) the mean across all except sNh‐ (excluding sNh‐). A comparison of *χ* ^2^ across models for each sequence is shown in Figure S10 (Supporting Information).

	H–J	M0	M1	M2
Overall mean	3.413	3.941	0.1475	1.949
excluding sNh‐	1.776	1.929	0.1466	1.026

We note that counterion condensation can lead to a substantial reduction in effective charge and varies across sequence and ionic strength (Figure S8, Supporting Information), consistent with our earlier observation with the Alexa dyes. As before, we also provide a comparison of the deviations of all four models from the measured data to highlight differences between models (Figure S9, Supporting Information). Moreover, Figure S10 (Supporting Information) compares the *χ* ^2^ values of the different models for each sequence. Finally, we provide a comparison of data against model M3, where *δ* is allowed to vary with ionic strength, for a given sequence (Figure S11, Supporting Information). M3 performs even slightly better than M2, confirming that our analysis is internally consistent. In fact, M3 improves the prediction for sCh compared to M2 (as discussed earlier), indicating that the salt dependence of *δ* can be important for this sequence. We reiterate that model M2 (equation 24 derived from data collected using the Alexa dyes) can be used to describe the data collected with Cy3B/CF660R dye pairs. This result, along with the observation that *δ* values are in a reasonable range (see Section S7.3, Supporting Information, for estimates) and that M3 provides improved or comparable performance as M2, indicates that *δ* is likely to have physical significance, even though we are currently not able to determine it from first principles.

### 3.4 Inferring Inter‐Residue Distance Maps

**Figure 5:**
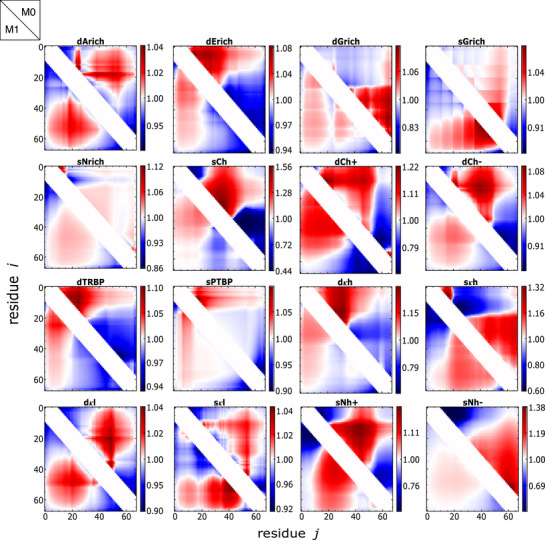
Inter‐residue distance maps (normalized by fits to homopolymer scaling; see Section S6, Supporting Information) for the 16 IDRs at 150 mM salt (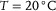), predicted using parameters (determined by matching end‐to‐end distances collected using Alexa 488/594) of model M1 (bottom triangle) and M0 (upper triangle). The maps clearly show a dependence on sequence, and differences between models are qualitatively consistent with those of Ref. [36].

So far, we have used single‐molecule FRET data to obtain mean square end‐to‐end distances $\left\langle R_{ee}^{2}\right\rangle$ across salt concentrations (and sequences) to determine the adjustable parameters of the models. However, our resulting models can also be utilized to predict the average distances $\left\langle R_{ij}^{2}\right\rangle$ between any two residues (*i*,*j*) in the chain, which are not available from the experimental data and would be difficult to obtain because only sequence separations greater than 30 or 40 residues can typically be probed with the Förster radii of the dye pairs available for single‐molecule FRET.^[^ ^33^, ^34^ ^]^ We present the distance maps predicted by the models in the normalized form $d_{ij}^{*}$ (see Section S6, Supporting Information, for details of calculation) using the parameters from M0 and compare them with the maps resulting from M1 (**Figure** **5**), where parameters were obtained by fitting data collected using the Alexa dyes.

The resulting distance maps indicate clear deviations from the uniform scaling of interresidue distance with sequence separation expected for simple homopolymers,^[^ ^20^ ^]^ and the regions of relative expansion (or compaction) are similar for the models M0 and M1. Moreover, features of distance maps (regions of expansion relative to homopolymer scaling in red and compaction in blue; see Section S6, Supporting Information, for details) for sequences dArich, sCh, dCh‐, sPTBP, dκh, sκh, sNh+, sNh‐ compare well with those computed from the coarse‐grained and atomistic simulations reported previously.^[^ ^36^ ^]^ These simulated maps were predicted after reweighting the ensemble (for atomistic simulation using ABSINTH^[^ ^58^ ^]^) or by fitting non‐electrostatic interaction parameters (for coarse‐grained simulation with the HPS model^[^ ^36^ ^]^) to match FRET efficiency data. Modest agreement is found for dGrich, dκl, sκl. However, dErich, sNrich, dCh+, dTRBP, and sGrich show clear deviations between the distance maps based on the simulations and the predictions based on our models. Interestingly, the same sequences (except dCh+) also exhibit differences in distance maps between coarse‐grained and atomistic simulations, indicating features in sequences that amplify differences in force fields and models. We note that theoretical predictions using M1 include dipolar interactions and partial ionization, which are not included in either simulation. On the other hand, non‐electrostatic interactions in M0 and M1 are modeled with the mean‐field assumption *ω*_2,*i*,*j*_ ≈ *ω*_2,*ee*_. Both factors can contribute to the observed discrepancies between theory and simulation. Additional experiments on the linker sequences would be needed to benchmark these predicted distance maps and thus assess the accuracy of theory, coarse‐grained simulations (HPS model), and atomistic simulations.^[^ ^36^ ^]^ Figure S12 (Supporting Information) shows the predicted distance maps using parameters derived from data collected with the Cy3B/CF660R dye pair. We also compared distance maps predicted from parameters obtained by fitting data collected using the two different dye pairs (Alexa dyes against Cy3B/CF660R dyes). These distance maps (Figure S13, Supporting Information) for a given sequence are very similar, with two notable exceptions for sCh and sNh‐. These differences most likely arise from the presence of the dyes, and are consistent with the effect of dyes seen in the measured chain dimensions for these two sequences (Figures 2 and 4).

### 3.5 Model With Ion Condensation is Required to Describe Inter‐Residue Distance Profiles

The preceding section highlights the ability of providing intra‐chain distances from our models. Motivated by this aspect, we tested our models' ability to describe inter‐residue distances for segments within the chain for two longer IDPs, ProTα (112‐113 residues) and Stm (598 residues). For Stm, we measured eight sets of distance pairs and for ProTα three distance pairs as a function of salt concentration. For this comparison, we use Cα‐Cα inter‐residue distances and model the dyes as one bead with an effective charge, neglecting the branched nature of the dyes not amenable to more detailed treatment in our current theoretical formalism (see Section S3, Supporting Information, for details of dye modeling). Since we have multiple sets of data for a given sequence, unlike for the linker sequences, we modify our parameter inference scheme from before. For a given sequence, we optimize for values of *p* and *δ* (similar to M1 for the 16 IDRs) that best describe the measured distances for all pairs. Moreover, residue pair (*i*,*j*)‐specific distances allow us to determine interaction strength values (*ω*_2,*i*,*j*_, defined in equation 20) individually (and do not assume they are identical for all segments) by fitting the high‐salt data for every segment probed. These parameters are expected to vary across residue pairs (*i*,*j*) due to variation in the non‐electrostatic two‐body interactions (*ω*_*m*,*n*_ in Equations 19 and 20) between different types of amino acids.

**Figure 6:**
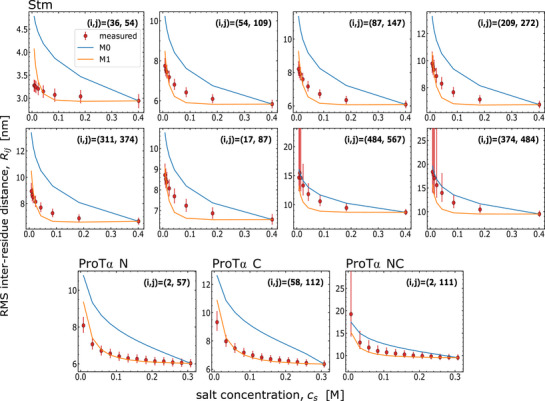
Inter‐residue distances of Stm and ProTα as a function of salt concentration, as measured by single‐molecule FRET (red), are described better by the sequence‐dependent condensation model (M1, orange), compared to the full ionization model (M0, blue). The best parameter values for Stm used here are $\tilde{p}=0.45,\delta =1.30$, and for ProTα $\tilde{p}=0.45,\delta =1.10$. Each plot shows a different inter‐residue pair as labeled by (*i*, *j*). Mean errors (chi‐square) between theory and experiment averaged across residue pairs, for Stm, are 27.680 for M0 and 2.803 for M1; for ProTα, they are 18.787 for M0 and 0.590 for M1. See Tables S4 and S5 (Supporting Information) for additional parameters, and Table S1 (Supporting Information) for the sequences. See Figure S14 (Supporting Information) for effective charges (degrees of ionization).The large error bars for Stm (484, 567), (374,484), and ProTα NC are a consequence of the low transfer efficiencies observed for these variants (see Methods, Supporting Information).

Both Stm and ProTα show a pronounced compaction with increasing salt concentration, as expected based on their large negative net charge and the low fraction of positively charged residues^[^ ^3^, ^37^ ^]^ (**Figure** **6**). We find that the ion‐condensation model M1 describes the salt‐dependent distances for Stm and ProTα reasonably well (Figure 6). The fitted values of *ω*_2,*i*,*j*_, exhibit modest variations from segment to segment, with Stm segments spanning values from 0.3 to 1.7, and ProTα segments values from 0.9 to 1.7 (Tables S4 and S5, Supporting Information). Remaining discrepancies between theory and experiment could be due to effects such as a variation of non‐electrostatic or dielectric mismatch parameters with ionic strength, which is neglected in the model. We also ignore local fluctuations in the dielectric mismatch. The full ionization model M0, in contrast, overestimates inter‐residue distances for both proteins, similar to the trend observed for the 16 linker IDRs, highlighting the important role of counterion condensation and the resulting reduction in effective charge. The inferred values of *p* ($\tilde{p}=0.45$) are within the range expected from our previous results (Section S7, Supporting Information). Furthermore, for both IDPs we obtain low values of *δ* (1.3 for Stm and 1.1 for ProTα) and an expanded chain at low salt, consistent with the trend seen in Figure 3 for the linker IDRs. We also note that predicting the distance between specific residue pairs is associated with minimizing the free energy with respect to three variables: distance and two effective charges (for positive and negative groups). Consequently, predicted distances between specific residue pairs will have associated with them a prediction of the effective degrees of ionization. A self‐consistent model must predict very similar values of these effective charges (degrees of ionization) that are global properties of the chain. As shown in Figure S14 (Supporting Information), these values are nearly identical, reflecting internal consistency of our model. As before, we also note that these effective charges gradually decrease with increasing salt concentration. As a further consistency check, we reanalyzed the 16 IDRs using Cα‐Cα distances with the single‐bead model for the dye. To ensure the robustness of our parameters, we kept the same values of *p* and *δ* from M2 and only fitted ω_2,*ee*_. Figures S15 and S16 (Supporting Information) show good agreement between the model and the data, supporting the robustness of the inferred parameters irrespective of the two slightly different models. For the Alexa 488/594 fluorophores, mean errors (chi‐squared) are 0.927 including all linker IDRs, and 0.147 when excluding sNh‐. Similarly, for Cy3B/CF660R, mean errors (chi‐square) are 2.244 including all sequences, and 0.956 excluding sNh‐.

## 4 Discussion

A large set of single‐molecule FRET experiments were performed to determine intrachain distances and global dimensions of IDPs with very different sequences and lengths; by varying the location of the FRET probes within the sequences; by using different FRET dye pairs; and by changing the salt concentration across a broad range to modulate the electrostatic interactions. These comprehensive sets of measurements were used to test different variants of analytically tractable polymer models that take into account the sequence‐specific charge distributions and to quantify several factors that affect electrostatic interactions in IDPs and influence their conformational distributions. Specifically, we used the data to test two competing models: (i) a model (M0) where charged amino acids are fully ionized, and (ii) a model (M1) allowing partial ionization due to condensation of oppositely charged counterions on the side chains. Predicting the extent of ion condensation is non‐trivial since it depends on the conformation which in turn depends on the condensation, requiring a self‐consistent treatment. M1 is such a self‐consistent analytical theory accounting for sequence effects (not just composition) to predict both chain dimensions and ionization. Although M0 also computes sequence‐dependent electrostatics within an analytical theory, we find that it is insufficient to explain the data. Specifically, M0 tends to overestimate the chain dimensions at low salt concentrations. The ion condensation model M1, on the other hand, can explain the data at different ionic strengths and outperforms M0. Different versions of ion condensation models were tested against the data to balance model complexity and agreement with experiment. It is possible to find a near‐predictive model (M2) where the dipole length resulting from the distance between counterion and side chain charge is treated as a shared parameter across all sequences, and the dielectric mismatch parameter is described by an empirical relation based on its correlation with the dimensions of the chains.

The better performance of the models taking into account ion condensation can be traced to two factors. First, the reduction in effective charge significantly alters the charge‐charge interactions (repulsive or attractive) compared to those in M0. Furthermore, new types of interactions, absent in M0, arise from the condensation of oppositely charged ions forming dipoles on the chain, which can contribute to intrachain interactions. Together, these two effects alter the sequence‐dependent interactions and the chains' response to salt concentration and consequently their conformational distributions. All these effects, and their sequence and solution dependence, are self‐consistently treated in model M1. This self‐consistent model can also predict – although not seen in the current set of sequences – first order phase transitions in charge and chain dimensions space by varying the Bjerrum length (inversely related to temperature) and salt for some sequences, as seen in our previous work.^[^ ^28^ ^]^

At present, we ignore the patterning effects from uncharged amino acids and their response to salt concentration. Our analytically tractable model also ignores the branched nature of the dyes attached to the chain via flexible linkers.^[^ ^36^ ^]^ These and other subtle conformational restrictions due to rotation and limited bond stretching, neglected in the theory, can be captured in simulations. Another assumption of the model is that charges of one type have the same degree of ionization, thus ignoring local differences in counterion condensation. Furthermore, we neglect spatial fluctuations of the dielectric constant and assume a mean‐field value for a given sequence. Moreover, we neglect variation of the dielectric constant and hydrophobicity (affecting *ω*_2_) with salt concentration. These effects may further modify the degree of condensation and may be responsible for the disagreement of the theory with one of the sequences we investigated experimentally.

The ability to describe a wide range of measurements on different disordered proteins with polymer models that take into account counterion condensation supports the need to further investigate the role of counterions for the interactions within and between IDPs, often ignored in computational studies of IDPs at present, especially in coarse‐grained models, and its impact on IDP conformation. Further directions to explore would be the effect of divalent ions, particularly for poly‐acidic IDPs, which would be particularly pertinent for understanding the role and regulation of IDPs in the cellular milieu. Accounting for this effect may also provide insights to the role of electrostatics in IDP function.^[^ ^59^ ^]^ The model presented here will be of practical use in such predictions due to its analytical and high‐throughput nature and the ability to generate new insights and hypotheses. For large IDPs, such as Stm, simulations remain challenging, especially at an atomistic level with explicit solvent, where explicit ion effects can be included.^[^ ^60^ ^]^ An interesting aspect will be to extend the current theory to include an explicit treatment of protonation equilibria,^[^ ^51^, ^61^ ^]^ especially for solutions at low salt concentrations and in pH ranges close to the *pK*_*a*_ values of ionizable amino acid residues. Another interesting extension of the theory would be to include the contribution of salting‐out effects or increased hydrophobicity at very high salt concentrations in the molar range.^[^ ^23^ ^]^ Analytical models like the ones we present here will continue to make an important contribution to the multi‐scale modeling of disordered proteins. The predicted degree of ionization might be used to modify charge states in coarse‐grained simulations. Finally, our work addresses the long‐standing question of counterion condensation in polymer physics mainly discussed for homopolymers. Our formalism extends the investigation to heteropolymers, highlighting the rich physics of the problem and stimulating future studies in natural and synthetic polymers.

## Conflict of Interest

The authors declare no conflict of interest.

## Author Contributions

M.P., A.H., M.W., and A.C. contributed equally to this work. M.P., A.H., M.W., A.C., S.K., A.Sor., A.O., B.S., and K.G. conceptualized the project, with M.P., A.H., A. Sor., B.S., K.G. as lead; M.P, A.H., A.C., N.L., J.H., D.N., B.S., K.G. participated in formal analysis, with M.P., A.H., A.C. as lead; M.P., A.H., M.W., A.C., A.Sor., A. Sot., S.K., N.M., N.L., J.H., M.L., B.S., K.G. participated in investigation; M.P., A.H., M.W., A.C., J.H., D.N., B.S., K.G. participated in methodology development; M.P., N.L., D.N., participated in software development, with M.P. as lead; M.P., A.H., A.C., B.S., K.G. contributed to visualization, with M.P. and A.C. as lead; M.P., M.W., A.C., A.O., B.S., K.G. were responsible for funding acquisition, with B.S. and K.G. as lead; M.P., A.O., B.S., K.G. did project administration, with B.S. and K.G. as lead; M.P., A.H., A.Sor., A.O., B.S., K.G. contributed to supervision, with B.S. and K.G. as lead, M.P., B.S., K.G wrote the original draft, with K.G. as lead; M.P., A.H., M.W., A.C., S.K., N.M., A.O., B.S. and K.G. participated in reviewing and editing the final draft.

## Supporting information

Supporting Information

## Data Availability

The data that support the findings of this study are available from the corresponding authors upon reasonable request.
