# Supplementary material for: Mapping Charge Interactions in Intrinsically Disordered Proteins
Source: Adv Sci (Weinh). 2025 Nov 26;13(16):e14056. doi: 10.1002/advs.202514056 (PMC13042941; doi:10.1002/advs.202514056)
Supplement: Supplementary file 1 — Supporting Information [file ADVS-13-e14056-s001.pdf]

Supporting Information for
Mapping charge interactions in intrinsically disordered proteins

Michael Phillips[#] Andrea Holla[#] Magdalena Wojtas[#] Aritra Chowdhury[#] Andrea Sottini Sebastian L. B. König Natalie Mutter Nick Lamb Jonathan Huihui Monika Lopko Andrea Soranno Daniel Nettels Andrzej Ożyhar Benjamin Schuler Kingshuk Ghosh**

[#]These authors contributed equally to this work
Dr. M. Phillips
Department of Physics and Astronomy, University of Denver, Denver, CO, USA
Dr. A. Holla
Department of Biochemistry, University of Zurich, Zurich, Switzerland
Dr. M. Wojtas
Department of Biochemistry, University of Zurich, Zurich, Switzerland
Department of Biochemistry, Faculty of Chemistry, Wrocław University of Technology, Wrocław, Poland
Dr. A. Chowdhury
Department of Biochemistry, University of Zurich, Zurich, Switzerland
Dr. A. Sottini
Department of Biochemistry, University of Zurich, Zurich, Switzerland
Dr. S. L. B. König
Department of Biochemistry, University of Zurich, Zurich, Switzerland
N. Mutter
Department of Biochemistry, University of Zurich, Zurich, Switzerland
N. Lamb
Department of Physics and Astronomy, University of Denver, Denver, CO, USA
Dr. J. Huihui
Department of Physics and Astronomy, University of Denver, Denver, CO, USA
Dr. M. Lopko
Department of Biochemistry, Faculty of Chemistry, Wrocław University of Technology, Wrocław, Poland
Prof. Dr. A. Soranno
Department of Biochemistry, University of Zurich, Zurich, Switzerland Department of Biochemistry and Molecular Biophysics, Center for Biomolecular Condensates, Washington University in St. Louis, St. Louis, MO, United States
Dr. D. Nettels
Department of Biochemistry, University of Zurich, Zurich, Switzerland
Prof. Dr. A. Ożyhar
Department of Biochemistry, Faculty of Chemistry, Wrocław University of Technology, Wrocław, Poland
Prof. Dr. B. Schuler
Department of Biochemistry and Department of Physics, University of Zurich, Zurich, Switzerland
Email address: schuler@bioc.uzh.ch
Prof. Dr. K. Ghosh
Department of Physics and Astronomy, University of Denver, Denver, CO, USA
Email address: kingshuk.ghosh@du.edu

S1 Protein purification and labeling

The disordered linker sequences from RNA-binding proteins (see Table S1) encoded in pET-20b(+) expression vectors were expressed in *E. coli* and purified as described by Holla et al. [1]. Briefly, linker constructs were expressed as fusion constructs with N- and C-terminal fusions of a His_6-tag and a GB1-His_6-tag, respectively (both of which were separated from the IDR of interest by a thrombin cleavage site) in *E. coli* Rosetta (DE3) cells (Merck Biosciences) and purified under denaturing conditions (6M guanidinium chloride) using immobilized metal ion affinity chromatography (IMAC); dialysis to non-denaturing conditions followed by thrombin-induced tag cleavage, concentration using 3-kDa molecular weight cut-off centrifugal concentrators, reduction and a final purification using reversed-phase high performance liquid chromatography (RP-HPLC) on a C18 column (Reprosil Gold 200, Dr. Maisch GmbH) using a water/acetonitrile gradient yielded the linkers, which were lyophilized and used for labeling. The linkers were labeled with AlexaFluor 488/AlexaFluor 594 or Cy3B/CF660R as described previously using a sequential labeling approach in which the lyophilized reduced proteins were reacted with substoichiometric (molar ratio 0.7) Alexa 488 or Cy3B (donor dyes) maleimides, followed by separation of the donor-labeled species using RP-HPLC on a C18 column (Reprosil Gold 200, Dr. Maisch GmbH), which was subsequently labeled with a stoichiometric excess ($\tilde{3}$-fold molar excess) of Alexa 594 (for Alexa 488-labeled samples) or CF660R maleimide (for Cy3B-labeled samples); purification of the double-labeled species containing donor and acceptor and labeling permutations (where possible) was achieved with a subsequent final RP-HPLC step.

Stm variants containing pairs of cysteine residues instead of alanine were prepared. Alanine codons were replaced by cysteine codons by site-directed mutagenesis using a PCR-based method described by Ko and Ma[2]. In the first stage, the Stm gene was amplified into two separate PCR fragments using two pairs of anchor and mutagenic primers. The anchor primers contained restriction sites for subcloning the mutated gene into corresponding sites of a pQE-80L (Qiagen, Germany) vector derivative generated in our laboratory without His-tag. The forward and reverse anchor primers contained BamHI and HindIII restriction sites, respectively, and the two mutagenic primers contained the desired mutation near the recognition site of the BsmBI restriction enzyme. After digestion of the two PCR fragments with the appropriate enzymes, both fragments were ligated together and into the vector to generate the construct with the mutated gene. To introduce a second mutation, the entire procedure was repeated using the previously obtained mutant as the template for PCR. Alternatively, when possible, the second mutation was introduced during the first stage using a mutated anchor primer. The DNA sequences of all double mutants were confirmed by sequencing.

Double cysteine variants of Starmaker without the signal peptide (Stm variants; see Table S1 for sequences) were purified as wild-type Stm (WT Stm) following Kaplon et al. [3]. Untagged Stm variants, cloned into the pQE-80L vector (Qiagen, Germany), were overexpressed in BL21(DE3) pLysS *E. coli* cells (Novagen, Germany) and purified to homogeneity using a three-step protocol: fractionation with solid $(NH_4)_2SO_4$, gel filtration, and hydroxyapatite chromatography. The samples were then reduced, concentrated, and further purified by RP-HPLC on a Vydac 214 TPC4 column using a water/acetonitrile gradient, then lyophilized for subsequent labeling. For labeling, the Stm pellet was diluted in 200 µL of 100 mM phosphate buffer (pH 7.0). The amount of free sulfhydryl groups in Stm was estimated using 5,5′-dithiobis(2-nitrobenzoic acid) (DTNB). To obtain pure donor- and acceptor-labeled Stm, a sequential labeling procedure was used. The first fluorophore (AlexaFluor 488 maleimide or AlexaFluor 594 maleimide), dissolved in DMSO, was added to the Stm solution at a 1 :1 molar ratio and allowed to react for 1 h at room temperature. The reaction was quenched by adding 10 mM DTT. The labeled Stm species were purified by RP-HPLC on a Vydac 214 TPC4 column. In most cases, the single-labeled Stm could be separated from unlabeled and double-labeled species; the fraction containing single-labeled Stm was frozen and lyophilized. For labeling with the second dye (AlexaFluor 594 or AlexaFluor 488 maleimide), the single-labeled Stm was diluted in 60 µL of 100 mM phosphate buffer (pH 7.0). The protein concentration was estimated from the maximum absorbance of the first dye, assuming single labeling. The second dye was then added in five- to ten-fold molar excess and allowed to react at room temperature for 1 h to 2 h. The reaction was terminated by adding 2 µL 2-mercaptoethanol. Finally, the double-labeled Stm was purified by RP-HPLC on a Vydac 214 TPC4 column.

ProTα C (isoform 1, see Table S1 for the sequence) was purified as an N-terminally His_6-tagged construct (the tag was removed by HRV 3C cleavage) and labeled as described previously[4][5]. ProTα NC and ProTα N (isoform 1, see Table S1) were cloned into a pBAD-IntCBD-12His vector and purified as described previously; briefly, the constructs were overexpressed in *E. coli* BL21 A1 cells carrying C-terminal fusions of the intein-chitin-binding domain-His_{12}, purified under native conditions with IMAC, followed by thiol-induced tag cleavage, dialysis, and reverse IMAC to remove the cleaved tag. The tag-free protein was concentrated with 3 kDa molecular-weight cut-off centrifugal filters and used for fluorescence labeling without further purification[5]. In brief, the reduced protein (treated with 10 mM TCEP followed by buffer exchange with centrifugal filters) was simultaneously labeled with ∼6.5 fold molar excess of Alexa 488 and Alexa 594 maleimide. After quenching the reaction and removing excess dye by sequential buffer exchange with centrifugal filtration, the double-labeled protein was purified by RP-HPLC on a Sunfire C18 column (Waters).

S2 Single-molecule measurements and analysis

Single-molecule experiments were performed on freely diffusing molecules with a MicroTime 200 confocal single-molecule instrument (PicoQuant, Berlin, Germany) or a custom-built setup as described previously[1]. Depending on the fluorophore under investigation, different excitation sources were used. Alexa 488 was excited using an LDH-D-C-485 diode laser (PicoQuant GmbH), whereas Alexa 594 and Cy3B were excited with a supercontinuum fiber laser (SC-450-4, Fianium Ltd.) that was filtered with a z582/15 or HC543.5/2 band-pass filter, respectively (Chroma Technology). For CF660R excitation, an LDH-D-C-640 diode laser (PicoQuant GmbH) was used. All lasers were operated at a pulse repetition rate of 20 MHz, which allowed for the pulsed interleaved excitation (PIE) of donor and acceptor molecules[6]. Fluorescence photons were collected using a UplanApo 60x/1.20W objective (Olympus) and passed through an appropriate multiband mirror and a 100-Î¼m confocal pinhole. The photons were subsequently separated by polarization with a polarizing beam splitter and by wavelength with appropriate dichroic mirrors. Finally, after passing through optical band-pass filters, the photons were detected by avalanche photodiodes, and their arrival times were recorded using a HydraHarp 400 time-correlated single-photon counting system (PicoQuant) with a resolution of 16 ps.

Experiments were performed at a temperature of 22 °C using chambered cover slides (µ-Slide, ibidi). For the linker sequences, experiments employed approximately 100 pM labeled molecules in 20 mM KH_2PO_4/K_2HPO_4 buffer (pH 7.3) with different KCl concentrations, supplemented with 0.001 % Tween 20 and 10 mM DTT for the Cy3B/CF660R-labeled samples, or with 147 mM 2-mercaptoethanol for the Alexa dye-labeled samples. Measurements in ionic strength-matched Bis-Tris or Tris buffer with KCl showed minor differences in transfer efficiencies of less than 0.02 for sCh, sNh-, and dTRBP compared to the reported measurements in phosphate buffer. For Stm and ProTα (all Alexa 488- and Alexa 594-labeled samples), measurements were performed in 10 mM Tris-HCl buffer (pH 7.4) with different KCl concentrations, 0.01 % Tween 20, and 147 mM 2-mercaptoethanol.

Fluorescence signals were acquired from single molecules freely diffusing through the confocal volume, and transfer efficiency E and the stoichiometry ratio S were quantified from selected photon bursts, each burst representing a single molecule traversing the observation volume, according to

$$E = \frac{N_{AD}}{N_{DD} + N_{AD}}, \quad S = \frac{N_{DD} + N_{AD}}{N_{DD} + N_{AD} + N_{AA}},$$

where N_{DD} and N_{AD} are donor and acceptor photons after donor excitation,respectively, that are corrected for background signals, direct acceptor excitation, and differences in dye quantum yields or detector efficiencies. N_{AA} correspond to the acceptor photons after acceptor excitation, corrected for background and for relative donor/acceptor excitation intensities as described previously [7] [8]. For Stm and ProTα measurements, the correction factors were obtained from the PIE measurements as described previously[9][8]. Data analysis was performed with Fretica, a custom add-on for Mathematica (Wolfram Research), available at https://github.com/SchulerLab.

Transfer efficiency histograms obtained under different experimental conditions were fitted with a Gaus-

sian peak function (for linkers) or by Photon Distribution Analysis (PDA)[10][11] to extract the mean transfer efficiency $\langle E \rangle$. The RMS end-to-end inter-dye distances were then inferred by numerically solving

$$\langle E \rangle = \int_0^\infty P(r)\, E(r)\, \mathrm{d}r, \quad E(r) = \frac{R_0^6}{R_0^6 + r^6},$$

where $P(r)$ is the distance probability density function, and R_0 is the Förster radius of the chosen fluorophore pair (5.4 nm for Alexa 488/Alexa 594 and 6.0 nm for Cy3B/CF660R). An empirically modified self-avoiding random-walk model (SAW-ν) was employed for $P(r)$[12]. The length scaling exponent ν for the IDP segments probed was estimated assuming an equivalent of nine additional amino acids accounting for the fluorophores and their linkers[8, 12], and the RMS C_α distances were then calculated using that value of ν and the number of peptide bonds in the chain segment bracketed by the labeling positions[12, 13]. The origin of the large uncertainties in inter-dye distances for Stm and ProTα variants probing long and highly charged segments at low salt concentrations (Fig. 6) is a result of very low observed transfer efficiencies, E: If the value of E approaches or falls below the systematic uncertainty of 0.03, we can effectively only provide a lower bound on the distance; as a result, the upper error bars diverge.

Table S1: Sequences of all IDRs used.

Name	Sequence	Charge
**dArich**	GSGSCLPTGAEGRDSSKGEDSAEETEAKPAVVAPAPVVEAVSTPSAAFPSDATAEQGPILTKCTLGPR	−10/ + 4
**dErich**	GSGSCEGEMFGVMSEENSVVLSVEQPAELKEVADVSPPPTTRNHTIEMKPPLSAQQSESNPVCTLGPR	−10/ + 3
**dGrich**	GSGSCGPRTGLEGAGMAGGSGQQKRVFDGQSGPQDLGEAYRPLNHDGGDGGNRYSVIDRIQECTLGPR	−8/ + 6
**sGrich**	GSGSCSGGYGSERGGGYGSERGGGYGSERGGGYGSERGGGYGSQRSGGGYGGSQRSSYGSGSCTLGPR	−4/ + 6
**sNrich**	GSGSCLDQEDNNGPLLIKTANNLIQNNSNMLPLNALHNAPPMHLNEGGISNMRVNDSLPSNTCTLGPR	−5/ + 2
**sCh**	GSGSCKKAQPEMNDKDDNESGNEDAEENHDDEEDENEEEDRQVDQASKNKESKRKAQNKREDCTLGPR	−23/ + 11
**dCh +**	GSGSCQTPLKRIKVKTPGKSGAAAREGSVVSGTDGPTQTGKPERRKRLNPPKDKLIDMDDADCTLGPR	−8/ + 13
**dCh −**	GSGSCMGLPTGMEEKEEGTDESEQKPVVQTPAQPDDSAEVDSAALDQAESAKQQGPILTKHGCTLGPR	−13/ + 4
**dTRBP**	GSGSCLEPALEDSSSFSPLDSSLPEDIPVFTAAAAATPVPSVVLTRSPPMELQPPVSPQQSECTLGPR	−8/ + 1
**sPTBP**	GSGSCRPDLPSGDSQPSLDQTMAAAFGLSVPNVHGALAPLAIPSAAAAAAAAGRIAIPGLAGCTLGPR	−3/ + 2
**dκh**	GSGSCAMGGGPGPGTDFTSDQADFPDTLFQEFEPPAPRPGLAGGRPGDAALLSAAYGRRRLLCTLGPR	−7/ + 5
**sκh**	GSGSCKPRRLSKLRRSKKPADEENNAASQDPTVEATQERGQASEDPENAANNAKQAKPTSDDCTLGPR	−11/ + 11
**dκl**	GSGSCLPTGMDQKTTELMKVDEPVSTQETLPPVIKMEPEPVPITETPTDENARQQGPILTKHCTLGPR	−10/ + 5
**sκl**	GSGSCAPEGVFKLPAPPKEIKKSEKSEKSSDSINDKKEVESTTKTAATTTTTKKGTDNNTQFCTLGPR	−9/ + 11
**sNh +**	GSGSCPEEIETRKKDRKNRREMLKRTSALQPAAPKPTHKKPVPKRNVGAERKSTINEDLLPPCTLGPR	−8/ + 16
**sNh −**	GSGSCKKKLQDTPSEPMEKDPAEPETVPDGETPEDENPTEEGADNSSAKMEEEEEEEEEEEEECTLGPR	−26/ + 5
**Stm 36_54**	GSAPVSNNNGTDNDESAADQRHILTVQFNVGTPAPCDGDSVTTDGKDSAEKNECPGDSSDTTEKPGTTDGKDSAEQHGVTTDGKDEAEQHGVTTEGQDSAEKRGETDGAPDKPDTQNGTDDTDSDKETDASHHKTGDSDENKDKPSAEDHTDGNHAGKDSTDSKESPDTTDKPEGPDSDSAPDGDSASAEKTDSDHSPDEDANKSSTEADKDDTSDKDSSQTDEKHDSDASDKDEKHEDKDEKSDEKDSSKDSEDKSLEKSDKSDDGSNSEADEQKESVESKDHDSDSQDSDSAEKKEKHDDKDQDSSDTADSKDSDEDKDKDHSEQKDSEDHEHKEKHTKDKEEHKDSDEGKDDEDKSKSDEHDKDESESKEASKSDESEQEEKKDDKSDSDNSSRDSHSDSDSDSHSDSDSDSHSDSHSDSDSDSHSDSDSDSDSDSDSDSDSDSDSNSRDKEEKKDKSSESRDEDSSDSDSKSNSESSETAEEDTNDDKDSSVEKDKTDSSDSASVEANDSDDEHDDDSKDATPSSEDHTAEKTDEDSHDVSDDDDDIDAHDDEAGVEHGTDEASKPHQEPDHHDDTTHGSDDGSKTSMPISKLN	−6/ + 2
**Stm 54_109**	GSAPVSNNNGTDNDESAADQRHILTVQFNVGTPAPADGDSVTTDGKDSAEKNECPGDSSDTTEKPGTTDGKDSAEQHGVTTDGKDEAEQHGVTTEGQDSAEKRGETDGCPDKPDTQNGTDDTDSDKETDASHHKTGDSDENKDKPSAEDHTDGNHAGKDSTDSKESPDTTDKPEGPDSDSAPDGDSASAEKTDSDHSPDEDANKSSTEADKDDTSDKDSSQTDEKHDSDASDKDEKHEDKDEKSDEKDSSKDSEDKSLEKSDKSDDGSNSEADEQKESVESKDHDSDSQDSDSAEKKEKHDDKDQDSSDTADSKDSDEDKDKDHSEQKDSEDHEHKEKHTKDKEEHKDSDEGKDDEDKSKSDEHDKDESESKEASKSDESEQEEKKDDKSDSDNSSRDSHSDSDSDSHSDSDSDSHSDSHSDSDSDSHSDSDSDSDSDSDSDSDSDSDSNSRDKEEKKDKSSESRDEDSSDSDSKSNSESSETAEEDTNDDKDSSVEKDKTDSSDSASVEANDSDDEHDDDSKDATPSSEDHTAEKTDEDSHDVSDDDDDIDAHDDEAGVEHGTDEASKPHQEPDHHDDTTHGSDDGSKTSMPISKLN	−15/ + 5
**Stm 87_147**	GSAPVSNNNGTDNDESAADQRHILTVQFNVGTPAPADGDSVTTDGKDSAEKNEAPGDSSDTTEKPGTTDGKDSAEQHGVTTDGKDECEQHGVTTEGQDSAEKRGETDGAPDKPDTQNGTDDTDSDKETDASHHKTGDSDENKDKPSCEDHTDGNHAGKDSTDSKESPDTTDKPEGPDSDSAPDGDSASAEKTDSDHSPDEDANKSSTEADKDDTSDKDSSQTDEKHDSDASDKDEKHEDKDEKSDEKDSSKDSEDKSLEKSDKSDDGSNSEADEQKESVESKDHDSDSQDSDSAEKKEKHDDKDQDSSDTADSKDSDEDKDKDHSEQKDSEDHEHKEKHTKDKEEHKDSDEGKDDEDKSKSDEHDKDESESKEASKSDESEQEEKKDDKSDSDNSSRDSHSDSDSDSHSDSDSDSHSDSHSDSDSDSHSDSDSDSDSDSDSDSDSDSDSNSRDKEEKKDKSSESRDEDSSDSDSKSNSESSETAEEDTNDDKDSSVEKDKTDSSDSASVEANDSDDEHDDDSKDATPSSEDHTAEKTDEDSHDVSDDDDDIDAHDDEAGVEHGTDEASKPHQEPDHHDDTTHGSDDGSKTSMPISKLN	+18/ + 7
**Stm 209_272**	GSAPVSNNNGTDNDESAADQRHILTVQFNVGTPAPADGDSVTTDGKDSAEKNEAPGDSSDTTEKPGTTDGKDSAEQHGVTTDGKDEAEQHGVTTEGQDSAEKRGETDGAPDKPDTQNGTDDTDSDKETDASHHKTGDSDENKDKPSAEDHTDGNHAGKDSTDSKESPDTTDKPEGPDSDSAPDGDSASAEKTDSDHSPDEDANKSSTECDKDDTSDKDSSQTDEKHDSDASDKDEKHEDKDEKSDEKDSSKDSEDKSLEKSDKSDDGSNSECDEQKESVESKDHDSDSQDSDSAEKKEKHDDKDQDSSDTADSKDSDEDKDKDHSEQKDSEDHEHKEKHTKDKEEHKDSDEGKDDEDKSKSDEHDKDESESKEASKSDESEQEEKKDDKSDSDNSSRDSHSDSDSDSHSDSDSDSHSDSHSDSDSDSHSDSDSDSDSDSDSDSDSDSDSNSRDKEEKKDKSSESRDEDSSDSDSKSNSESSETAEEDTNDDKDSSVEKDKTDSSDSASVEANDSDDEHDDDSKDATPSSEDHTAEKTDEDSHDVSDDDDDIDAHDDEAGVEHGTDEASKPHQEPDHHDDTTHGSDDGSKTSMPISKLN	−27/ + 12
**Stm 311_374**	GSAPVSNNNGTDNDESAADQRHILTVQFNVGTPAPADGDSVTTDGKDSAEKNEAPGDSSDTTEKPGTTDGKDSAEQHGVTTDGKDEAEQHGVTTEGQDSAEKRGETDGAPDKPDTQNGTDDTDSDKETDASHHKTGDSDENKDKPSAEDHTDGNHAGKDSTDSKESPDTTDKPEGPDSDSAPDGDSASAEKTDSDHSPDEDANKSSTEADKDDTSDKDSSQTDEKHDSDASDKDEKHEDKDEKSDEKDSSKDSEDKSLEKSDKSDDGSNSEADEQKESVESKDHDSDSQDSDSAEKKEKHDDKDQDSSDTCDSKDSDEDKDKDHSEQKDSEDHEHKEKHTKDKEEHKDSDEGKDDEDKSKSDEHDKDESESKECSKSDESEQEEKKDDKSDSDNSSRDSHSDSDSDSHSDSDSDSHSDSHSDSDSDSHSDSDSDSDSDSDSDSDSDSDSNSRDKEEKKDKSSESRDEDSSDSDSKSNSESSETAEEDTNDDKDSSVEKDKTDSSDSASVEANDSDDEHDDDSKDATPSSEDHTAEKTDEDSHDVSDDDDDIDAHDDEAGVEHGTDEASKPHQEPDHHDDTTHGSDDGSKTSMPISKLN	−30/ + 14
**Stm 17_87**	GSAPVSNNNGTDNDESCADQRHILTVQFNVGTPAPADGDSVTTDGKDSAEKNEAPGDSSDTTEKPGTTDGKDSAEQHGVTTDGKDECEQHGVTTEGQDSAEKRGETDGAPDKPDTQNGTDDTDSDKETDASHHKTGDSDENKDKPSAEDHTDGNHAGKDSTDSKESPDTTDKPEGPDSDSAPDGDSASAEKTDSDHSPDEDANKSSTEADKDDTSDKDSSQTDEKHDSDASDKDEKHEDKDEKSDEKDSSKDSEDKSLEKSDKSDDGSNSEADEQKESVESKDHDSDSQDSDSAEKKEKHDDKDQDSSDTADSKDSDEDKDKDHSEQKDSEDHEHKEKHTKDKEEHKDSDEGKDDEDKSKSDEHDKDESESKEASKSDESEQEEKKDDKSDSDNSSRDSHSDSDSDSHSDSDSDSHSDSHSDSDSDSHSDSDSDSDSDSDSDSDSDSDSNSRDKEEKKDKSSESRDEDSSDSDSKSNSESSETAEEDTNDDKDSSVEKDKTDSSDSASVEANDSDDEHDDDSKDATPSSEDHTAEKTDEDSHDVSDDDDDIDAHDDEAGVEHGTDEASKPHQEPDHHDDTTHGSDDGSKTSMPISKLN	−16/ + 6

Continued on next page

Table S1 (continued)

Name	Sequence	Charge
**Stm 484_567**	GSAPVSNNNGTDNDESAADQRHILTVQFNVGTPAPADGDSVTTDGKDSAEKNEAPGDSSDTTEKPGTT<br>DGKDSAEQHGVTTDGKDEAEQHGVTTEGQDSAEKRGETDGAPDKPDTQNGTDDTDSDKETDASHHK<br>TGDSDENKDKPSAEDHTDGNHAGKDSTDSKESPDTTDKPEGPDSDSAPDGDSASAEKTDSDHSPDEDA<br>NKSSTEADKDDTSDKDSSQTDEKHDSDASDKDEKHEDKDEKSDEKDSSKDSEDKSLEKSDKSDDGSNSE<br>ADEQKESVESKDHDSDSQDSDSAEKKEKHDDKDQDSSDTADSKDSDEDKDKDHSEQKDSEDHEHKEKH<br>TKDKEEHKDSDEGKDDEDKSKSDEHDKDESESKEASKSDESEQEEKKDDKSDSDNSSRDSHSDSDSDSH<br>SDSDSDSHSDSHSDSDSDSHSDSDSDSDSDSDSDSDSDSDSNSRDKEEKKDKSSESRDEDSSDSDSKSNSESS<br>ETCEEDTNDDKDSSVEKDKTDSSDSASVEANDSDDEHDDDSKDATPSSEDHTAEKTDEDSHDVSDDDD<br>DIDAHDDEAGVEHGTDECSKPHQEPDHHDDTTHGSDDGSKTSMPISKLN	−38/ + 5
**Stm 374_484**	GSAPVSNNNGTDNDESAADQRHILTVQFNVGTPAPADGDSVTTDGKDSAEKNEAPGDSSDTTEKPGTT<br>DGKDSAEQHGVTTDGKDEAEQHGVTTEGQDSAEKRGETDGAPDKPDTQNGTDDTDSDKETDASHHK<br>TGDSDENKDKPSAEDHTDGNHAGKDSTDSKESPDTTDKPEGPDSDSAPDGDSASAEKTDSDHSPDEDA<br>NKSSTEADKDDTSDKDSSQTDEKHDSDASDKDEKHEDKDEKSDEKDSSKDSEDKSLEKSDKSDDGSNSE<br>ADEQKESVESKDHDSDSQDSDSAEKKEKHDDKDQDSSDTADSKDSDEDKDKDHSEQKDSEDHEHKEKH<br>TKDKEEHKDSDEGKDDEDKSKSDEHDKDESESKECSKSDESEQEEKKDDKSDSDNSSRDSHSDSDSDSH<br>SDSDSDSHSDSHSDSDSDSHSDSDSDSDSDSDSDSDSDSDSNSRDKEEKKDKSSESRDEDSSDSDSKSNSESS<br>ETCEEDTNDDKDSSVEKDKTDSSDSASVEANDSDDEHDDDSKDATPSSEDHTAEKTDEDSHDVSDDDD<br>DIDAHDDEAGVEHGTDEASKPHQEPDHHDDTTHGSDDGSKTSMPISKLN	−42/ + 12
**ProTα N**	GCDAAVDTSSEITTKDLKEKKEVVEEAENGRDAPANGNAENEENGEQEADNEVDEECEEGGEEEEEE<br>EEGDGEEEDGDEDEEAESATGKRAAEDDEDDDVDTKKQKTDEDDGA	−20/ + 5
**ProTα C**	GPSDAAVDTSSEITTKDLKEKKEVVEEAENGRDAPANGNAENEENGEQEADNEVDEECEEGGEEEEEE<br>EEGDGEEEDGDEDEEAESATGKRAAEDDEDDDVDTKKQKTDEDC	−32/ + 5
**ProTα NC**	GCDAAVDTSSEITTKDLKEKKEVVEEAENGRDAPANGNAENEENGEQEADNEVDEEEEEGGEEEEEEE<br>EGDGEEEDGDEDEEAESATGKRAAEDDEDDDVDTKKQKTDEDCGA	−53/ + 10

S3 Modeling the FRET Dyes

The properties of the fluorophores, especially their charge and finite size, should be included in the model to account for their effect on the observed dye-to-dye distance. Moreover, the charges from the terminal α-amino and α-carboxyl groups need to be accounted for. For the IDRs from RNA-binding proteins, we modeled the dyes as short sequences of residues that approximate the size and charge of the fluorophores. Dyes are attached to Cysteine residues near the terminals of the sequences, with small tails at each end (Table S1); each sequence begins with `GSGS` and ends with `TLGPR`. To account for the terminal charges, we replaced the first residue `G` $\rightarrow$ `K`, and the last residue `R` $\rightarrow$ `A`. Short sequences of 4 and 5 residues approximate the size of the dyes[8] for both dye pairs (Alexa 488/594 or Cy3B/CF660R, see Figure S1). Since the donor/acceptor labeling permutations are not well defined in all cases[1], we modeled both labeling permutations and found negligible differences in inter-dye distances. For simplicity, all results are presented with dyes modeled with the donor on the N- and the acceptor at the C-terminus, and we represent the charge content of each dye as a linear sequence of suitably charged residues. Alexa 488 was thus modeled as `EEKE`, and Alexa 594 as `EAEKE`; in combination with the amino acid tails and the terminal charges, we ultimately replaced `GSGS`$\rightarrow$`AEKE` at the N-terminus and `TLGPR`$\rightarrow$`EAEKE` at the C-terminus. Simliarly, Cy3B was modeled as `EKAA`, and CF660R as `EEKKE`; again combining with the terminal charges, we ultimately replaced `GSGS`$\rightarrow$`EKAA` at the N-terminus and `TLGPR`$\rightarrow$`EEKKE` at the C-terminus.

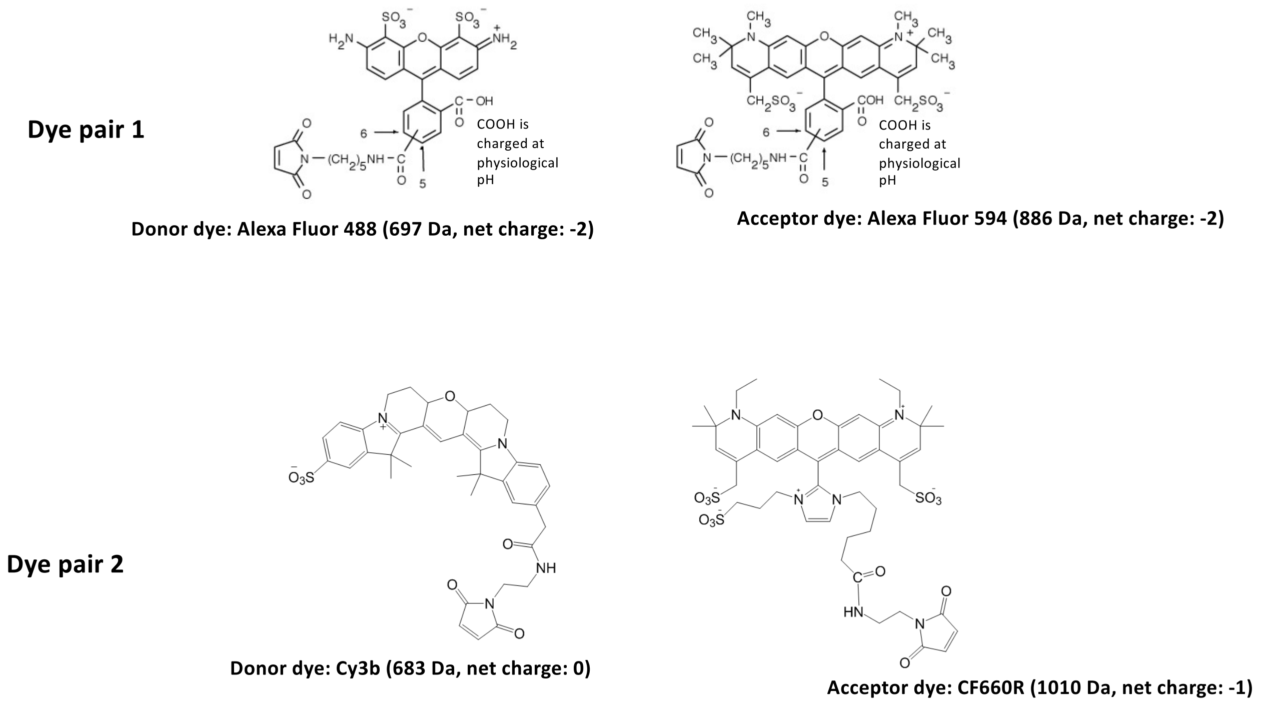

Figure S1: FRET dye pairs used for labeling of the linker sequences. For Stm and ProTα, only dye pair 1 (Alexa 488/594) was used. To approximate their size and charge, we modeled the first of each pair (left) as a 4-residue segment at the N-terminus, and the second of each pair (right) as a 5-residue segment at the C-terminus.

For long and highly charged sequences, such as Stm and ProTα, the dyes have a minor influence on chain properties. Different segments of these sequences were probed by placing Cys (`C`) for dye labeling at specific positions along the chain, which precludes the possibility of modeling the size of the fluorophores in the detail used for linker sequences; instead, we modeled the dyes by simply replacing Cys by single residues of appropriate net charge. Due to their large lengths and charge, we also omitted adjustments from terminal charges as their effects would be negligible. Only the dye pair Alexa 488/594 was used in

both Stm and ProTα, so we ultimately just replaced each C$\rightarrow$X with charge -2. For the sake of comparison between the two approaches of representing the dyes, we also modeled the dyes in the linker sequences without including additional residues accounting for their size, using only the net charge of the dyes as single residues at the Cys labeling positions, but keeping the terminal charge adjustments as described above. For Alexa 488/594, we replaced each C by a residue X with charge -2. For Cy3B/CF660R, we left the first C unchanged since Cy3B is net neutral, and we replaced the second C by D since CF660R has net charge -1. Note that full sequences of Table S1 are used in the inter-residue theory for RMS distances $\sqrt{\langle R_{ij}^2 \rangle}$, not just the segments between dyes at locations (i, j), because the theory accounts for context provided by the parts of the sequence outside of the probed segments.

We also note that charge assignments of residues in all sequences are K,R $\rightarrow +1$ and E,D $\rightarrow -1$. In most sequences, we consider Histidine as neutral. This approximation is justified since the linker IDRs have at most 2 His residues, and ProTα has zero. However, Stm has 32 His residues so for those sequences we use the charge assignment H $\rightarrow +0.5$.

S4 Details of free energy

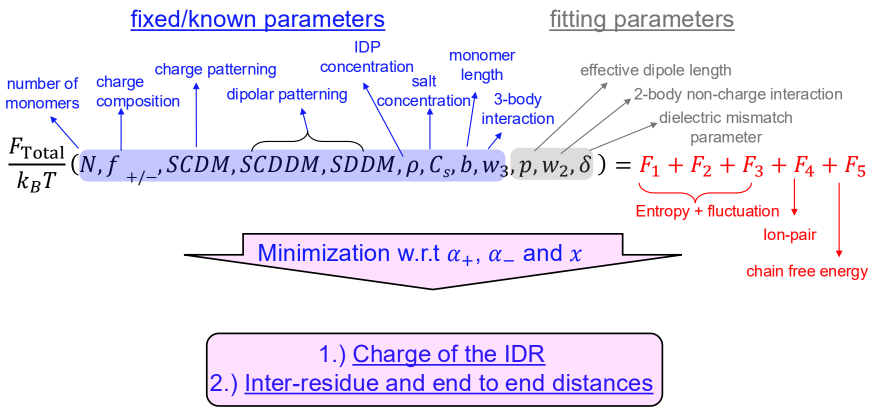

Figure S2: A schematic of the free energy and its optimization. The different contributions to the total free energy are depicted in red. The free energy is minimized with respect to effective ionization and swelling ratio to determine optimal charge and distance. Several parameters are known and fixed (in blue), and only the parameters in gray are inferred from fitting the experimental data.

S5 Higgs-Joanny Model

We describe here the specific form of the Higgs-Joanny model (H-J) used in main text Figure 2 and 4, as well as SI Figures S4 and S9. We present the H-J model using the same swelling/compaction variable x as employed in the sequence patterning models (M0, M1, M2), related to RMS end-to-end distance by $\langle R_{ee}^2 \rangle = N\ell bx$. This notation differs from the original formulation, which also defines a swelling/compaction parameter but in a different way[14].

For a sequence with N residues, N_+ with positive charge (K, R) and N_- with negative charge (E, D), and the rest neutral (either hydrophobic or polar), the fractions of each charge type are $f_+ = N_+/N$ and $f_- = N_-/N$. For the Debye length, κ^{-1} in Debye-Hückel screening, we use $\kappa^2 = 8\pi\ell_B c_s$, where ℓ_B is the Bjerrum length and c_s is the salt concentration (equivalent to ionic strength in our case of monovalent ions).

Essentially, the H-J model is equivalent to a self-avoiding walk model with the two-body interaction modulated by electrostatic effects. The bare two-body non-electrostatic interaction is ω_2. The two-body interaction is then combined with electrostatic effects due to the polyelectrolyte and polyampholyte properties of the sequence related to net charge and total charge, respectively (see Ref. [14] for details). The total two-body interaction strength is given by

$$v_2 = \omega_2 + \frac{4\pi\ell_B(f_+ - f_-)^2}{(\ell b)^{3/2}\,\kappa^2} - \frac{\pi\ell_B^2(f_+ + f_-)^2}{(\ell b)^{3/2}\,\kappa} \tag{S1}$$

Note that we use a geometric mean between bond length ($b = 3.8$ Å) and Kuhn length ($\ell = 8$ Å) as the single length scale in the H-J model, $\sqrt{\ell b} \approx 5.5$ Å, to reconcile the original H-J formulation with the relation $\langle R_{ee}^2 \rangle = N\ell bx$. We note that the Higgs-Joanny model is strictly applicable only in the high-salt limit ($\kappa^2\ell b \gg 1$) and formally diverges as the salt concentration approaches zero. This necessitates the introduction of a three-body repulsive interaction to maintain physical results at low salt (52 mM), as described below.

Although the original formulation of Higgs and Joanny is in terms of an equation of state, we integrate that to obtain the effective free energy. With the above two-body interaction strength, the H-J model adopts the same form as our model M0 apart from the sequence-dependent electrostatic contribution, i.e. it reads as the main text Eq. 6 with $Q = 0$. Two-body interactions are included in the term Ω in the same way as in the main text Eq. 12, replacing $\omega_{2,ee} \to v_2$. Based on that, we write the weight of two-body interactions here with the double summation

$$\Omega_0 = \frac{1}{N}\sum_{m=2}^{N}\sum_{n=1}^{m-1}(m-n)^{-1/2} \tag{S2}$$

The original H-J model does not include any three-body interaction, but we add it here because it is needed to prevent unphysical chain collapse in the strong polyampholyte limit, which applies for a subset of the linker IDRs. The three-body term has the same form as in main text Eq. 6, with weight B given by main text Eq. 7.

The effective free energy for the H-J model as used in this work is then

$$\beta F_{HJ} = \frac{3}{2}(x - \ln(x)) + v_2\Omega_0\left(\frac{3}{2\pi x}\right)^{3/2} + \frac{\omega_3 B}{2}\left(\frac{3}{2\pi x}\right)^3 \tag{S3}$$

As with our model M0, the above free energy for the H-J model is minimized to obtain x, and thus $\langle R_{ee}^2 \rangle$, at each salt concentration. Note that differentiating Eq. S3 and setting $\omega_3 = 0$ in the limit of large N recovers the original H-J formulation of Ref. [14], Eq. 4.12.

S6 Generating normalized distance maps

The overall theoretical approach for calculating distance maps is explained in main text Section 2.2. Briefly, we have a free energy that depends on sequence (charge patterning) and effective charge, which determines the inter-residue compaction or swelling through the chain factor x_{ij}, for each residue index pair (i, j). We then obtain the actual RMS distance from the relation $\langle R_{ij}^2 \rangle = x_{ij}\, |i-j| b\ell$, using the bond length $b = 3.8$ Å and Kuhn length $\ell = 8.0$ Å.

It is possible to construct inter-residue profile maps using the RMS distance directly, $\sqrt{\langle R_{ij}^2 \rangle}$. However, depending on the sequence, the features of such a direct map are generally dominated by homopolymer scaling, i.e. $\sqrt{\langle R_{ij}^2 \rangle} \propto |i-j|^\nu$, where ν is a scaling exponent. In particular, differences between sequences of the same length can be very subtle in the direct mapping, even though the theory includes sequence dependence. Further, differences between models (e.g. for the linker IDRs, the full ionization model, M0, and the fitted effective charge model with counterion condensation, M1) can appear negligible based on direct maps.

For better comparison between sequences and between models, and to delineate subtle features of the maps, we therefore sought a way to normalize the distance maps, focusing on deviations from the homopolymer model as was done in the previous work [1, 15, 16]. We term the normalized distance map d_{ij}^*. This normalization divides the homopolymer contribution as

$$
\begin{aligned}
d_{ij}^* &= \frac{\sqrt{\langle R_{ij}^2 \rangle}}{A_0 |i-j|^\nu} \\
&= \frac{\sqrt{b\ell x_{ij}}\; |i-j|^{0.5-\nu}}{A_0}
\end{aligned}
\tag{S4}
$$

where the prefactor A_0 and exponent ν must be determined from a fitting procedure. As eq. S4 makes clear, the normalization is not simply mapping x_{ij}.

The fitting procedure to determine A_0 and ν first requires the RMS distance $\sqrt{\langle R_{ij}^2 \rangle}$ averaged across residues with fixed index separation $\Delta = |i-j|$,

$$
R_\Delta = \frac{1}{N-\Delta} \sum_{i=1+\Delta}^{N} \left[\sqrt{\langle R_{ij}^2 \rangle} \right]_{j=i-\Delta}
\tag{S5}
$$

We then use a least-squares algorithm to fit eq. S5 to the homopolymer form, $R_\Delta \approx A_0\, \Delta^\nu$. The best fit values of A_0 and ν (in the range $\nu \in [0.3, 0.7]$) are then used in eq. S4 to obtain d_{ij}^*.

This procedure was employed for all sequences and models of interest; the resulting normalized maps of d_{ij}^* are presented in main text Figure 5, and SI Figure S12 and S13.

S7 Estimates of key model parameters

S7.1 Dipole Length

The physical separation, p, of a condensed ion from the side chain ion it interacts with is also the length of dipoles in charge-dipole and dipole-dipole interactions. We estimate p based on properties of proteins and their constituent amino acids to apply our model and assess best fit parameters. Charged side chains contain either a carboxylate group (Glutamic acid and Aspartic acid), a protonated amino group (Lysine), or a guanidinium group (Arginine). We consider the ions in the system to be dominated by salt ions, i.e. K^+ and Cl^-, rather than the smaller H^+. The distance separating a negative side chain ion O^- from its condensed counterion K^+ is estimated by the sum of half bond lengths of C-O and K-Cl. Likewise, the distance separating a positive side chain ion from its counterion is the sum of half bond lengths from N-H and K-Cl. Using values from NIST[17] to two significant figures, the bond length of C-O is 1.2 Å, N-H is 1.0 Å, and K-Cl is 2.7 Å. Sums of half bond lengths are then 1.95 Å and 1.85 Å. Taking the mean, we arrive at $p \approx 1.9$ Å. We obtain non-dimensional values by dividing by the bond length between amino acids, $b = 3.8$ Å, giving $\tilde{p} = p/b \approx 0.5$. The range of these values gives approximately $0.48 < \tilde{p} < 0.51$. A second independent estimate of dipole length was established previously based on the Avogadro software (see SI of ref. [18]). The range of values from Avogadro gives approximately $0.48 < \tilde{p} < 0.65$. Based on these considerations and our previous estimates including NaCl as ionic contributions [18], we expect the separation to be approximately in the interval $\tilde{p} \in [0.45, 0.65]$.

S7.2 Protein Concentration

FRET experiments were performed on single molecules, corresponding to very low protein concentrations of approximately 1 pM. Theoretical models with ion condensation involve division by the concentration, which can present numerical difficulties when it is too low. We tested several values and found that results are insensitive to the specific concentration so long as it is significantly less than 1 mM. Ultimately, we used the concentration $\rho = 1\,\mu$M in all theoretical formulations, for all sequences; this is low enough to mimic experimental reality and high enough to support numerical minimization of the free energy. In reduced (non-dimensional) units, the value is $\tilde{\rho} = \rho b^3 = 3.304 \times 10^{-8}$.

S7.3 Dielectric mismatch

Another important parameter in our model is the dielectric mismatch, defined as the ratio of bulk permittivity of water to the local permittivity near a protein, $\delta = \epsilon/\epsilon_l$. To assess our fitted values, we refer to an estimate of local permittivity from a different model fit to data, $\epsilon_l = 45 \pm 13$ [19]. With the standard value for water, $\epsilon = 80$, this suggests an expected ratio $\delta \approx 1.8$. Accounting for reported uncertainty in ϵ_l leads to the estimated range $\delta \in [1.3, 2.5]$.

S8 Parameter values for linkers IDRs from various models

We report the final values of physical parameters for each model used.

The three-body interaction parameter ω_3 was assumed to be 0.1 throughout based on a previous estimate using all-atom simulations [20]. Recent work [21] based on coarse-grained simulations has also shown $\omega_3 = 0.1$ to be a reasonable choice and $\omega_3 = 0.2$ to be optimal. The full ionization model, M0, has only a single free parameter — the two-body interaction strength $\omega_{2,ee}$ that was determined by matching the high-salt data. This parameter can be obtained by a simple algebraic equation (see Appendix in Reference [22]) derived by setting the derivative of free energy to zero, the criterion for the minimum in free energy. Since derivatives can be zero for both the maximum and minimum of the free energy, it is important to ensure that the free energy profile with the inferred $\omega_{2,ee}$ indeed yields a free energy minimum at the chain dimensions that match the experimentally measured value. For all cases, this additional self-consistency check was performed.

The ion condensation models, M1 and M2, differ only in how the dielectric mismatch parameter, δ, is determined. In M1, δ is determined by fitting the end-to-end distance vs salt data, constrained to the interval $\delta \in [1.0, 2.7]$, with $\omega_{2,ee}$ still set to match the high-salt measurement, and the other parameters fixed. In M2, δ is determined from the best linear fit with low-salt end-to-end distance. This line itself is determined by adjusting slope and intercept at each iteration, setting the value of δ for each sequence based on that iteration's line (while ensuring $\delta \geq 1$), and calculating χ^2 across all sequences (except the outlier sNh−); iterations continued until the total χ^2 was minimized. The dipole length was kept fixed at $\tilde{p} = 0.66$ for both M1 and M2 — this is the optimal dipole length determined by global search, repeating the fitting process of M1 for many values of $\tilde{p}$ and selecting the one yielding minimum total χ^2 from all linker IDRs. We use a standard definition of χ^2, for measured data with asymmetric error bars. We write a given measurement of RMS end-to-end distance as R_m and the corresponding upper and lower uncertainties as σ_+ and σ_-, respectively. The corresponding theoretical prediction is then R_p. Note, all these values depend on salt concentration, c_s. For a given sequence S, we obtain a mean chi-squared error across salt concentrations as

$$\chi^2(S) = \frac{1}{N_s} \sum_{\{c_s\}} \frac{(R_p - R_m)^2}{\sigma_+ \sigma_-} \tag{S6}$$

where N_s is the number of different salt concentrations for that sequence. A total value is obtained by simply summing over all sequences under consideration: $\chi^2_{total} = \sum_{\{S\}} \chi^2(S)$. (A mean over sequences can be used alternatively, but it makes no difference for optimization.) This approach was also used for fitting the results from Stm and ProTα, simply using inter-residue distances and their uncertainties instead of end-to-end.

Below we provide parameter values for different models, specified as table columns for comparison. Sequences with different dye pairs, Alexa 488/594 and Cy3B/CF660R, are in separate tables.

**Sequence**	**M0** $\omega_{2,ee}$	**M1** $\omega_{2,ee}$	**M2** $\omega_{2,ee}$	**M1** δ	**M2** δ
dArich	0.458	0.694	0.747	1.170	1.324
dErich	0.658	0.947	0.983	1.186	1.279
dGrich	-0.105	0.022	0.059	1.393	1.572
sGrich	-0.426	-0.350	-0.321	1.558	1.778
sNrich	0.053	0.167	0.172	1.423	1.467
sCh	-0.362	0.857	0.846	1.558	1.547
dCh+	0.162	0.380	0.376	1.630	1.619
dCh−	0.409	1.024	0.925	1.504	1.310
dTRBP	0.349	0.551	0.653	1.000	1.308
sPTBP	-0.077	-0.052	-0.011	1.000	1.535
dκh	-0.137	-0.013	0.008	1.505	1.629
sκh	0.147	0.411	0.394	1.694	1.650
dκl	1.128	1.324	1.354	1.176	1.272
sκl	0.620	0.761	0.748	1.473	1.434
sNh+	0.230	0.563	0.555	1.572	1.555
sNh−	-0.437	2.660	1.731	1.870	1.255

Table S2: Parameters resulting from each model (M0, M1, M2). Ion condensation models (M1,M2) used a fixed dipole length, $\tilde{p} = 0.66$. The proteins here were labeled with Alexa 488/594.

**Sequence**	**M0** $\omega_{2,ee}$	**M1** $\omega_{2,ee}$	**M2** $\omega_{2,ee}$	**M1** δ	**M2** δ
dArich	0.955	1.105	1.106	1.325	1.328
dErich	1.193	1.375	1.364	1.339	1.294
dGrich	0.059	0.073	0.150	1.000	1.533
sGrich	-0.237	-0.221	-0.153	1.000	1.661
sNrich	0.468	0.474	0.497	1.000	1.407
sCh	0.395	1.811	1.398	2.034	1.561
dCh+	0.428	0.633	0.652	1.399	1.449
dCh−	0.742	1.213	1.078	1.740	1.352
dTRBP	1.610	1.707	1.751	1.000	1.230
sPTBP	0.234	0.228	0.242	1.000	1.447
dκh	-0.012	0.071	0.059	1.715	1.631
sκh	0.744	1.089	0.897	2.000	1.467
dκl	1.843	1.898	1.943	1.000	1.251
sκl	1.110	1.130	1.205	1.000	1.340
sNh+	1.245	1.595	1.578	1.330	1.299
sNh−	0.079	3.356	1.993	2.700	1.342

Table S3: Parameters resulting from each model (M0, M1, M2). Ion condensation models (M1,M2) used a fixed dipole length, $\tilde{p} = 0.66$. The proteins here were lableled with Cy3B/CF660R.

S9 Non-Electrostatic Interactions for Stm and ProTα

We report the values of two-body non-electrostatic interaction strengths, $\omega_{2,ij}$, for Stm and ProTα. They depend on probed residue indices (i,j) as described in main text Section 2.2 and equation 19, 20 and 21. They were obtained by matching the high-salt data point of each inter-residue segment, for each sequence. We include results from the full ionization model (M0), as well as the effective charge counterion condensation model (M1). In all cases, the three-body non-electrostatic interaction strength was fixed at $\omega_3 = 0.1$.

**Residue Indices** (i,j)	**M0** $\omega_{2,ij}$	**M1** $\omega_{2,ij}$
(36,54)	-0.812	0.322
(54,109)	-0.441	0.530
(87,147)	-0.581	0.490
(209,272)	-0.879	0.810
(311,374)	-0.870	0.771
(17,87)	-0.201	0.540
(484,567)	-1.172	1.718
(374,484)	-1.185	0.997

Table S4: Two-body interaction strengths for each segment (pair of residue indices shown in left column) as determined by matching the high-salt data, for Stm. These are parameter values for both models (M0 and M1) shown in Figure 6 of the main text. In M1, additional parameters are $\tilde{p} = 0.45$, $\delta = 1.30$

**Residue Indices** (i,j)	**M0** $\omega_{2,ij}$	**M1** $\omega_{2,ij}$
(2,57)	-1.987	0.935
(58,112)	-5.425	1.433
(2,111)	-3.159	1.741

Table S5: Two-body interaction strengths for each segment (pair of residue indices shown in left column) as determined by matching the high-salt data, for ProTα N, C, NC from top to bottom (see Table S1). These are parameter values for both models (M0 and M1) shown in Figure 6 of the main text. In M1, additional parameters are $\tilde{p} = 0.45$, $\delta = 1.10$

S10 Additional plots for linker IDRs

The figure below shows predicted degrees of ionization from models M1 and M2 using parameters inferred by fitting data collected using Alexa dyes.

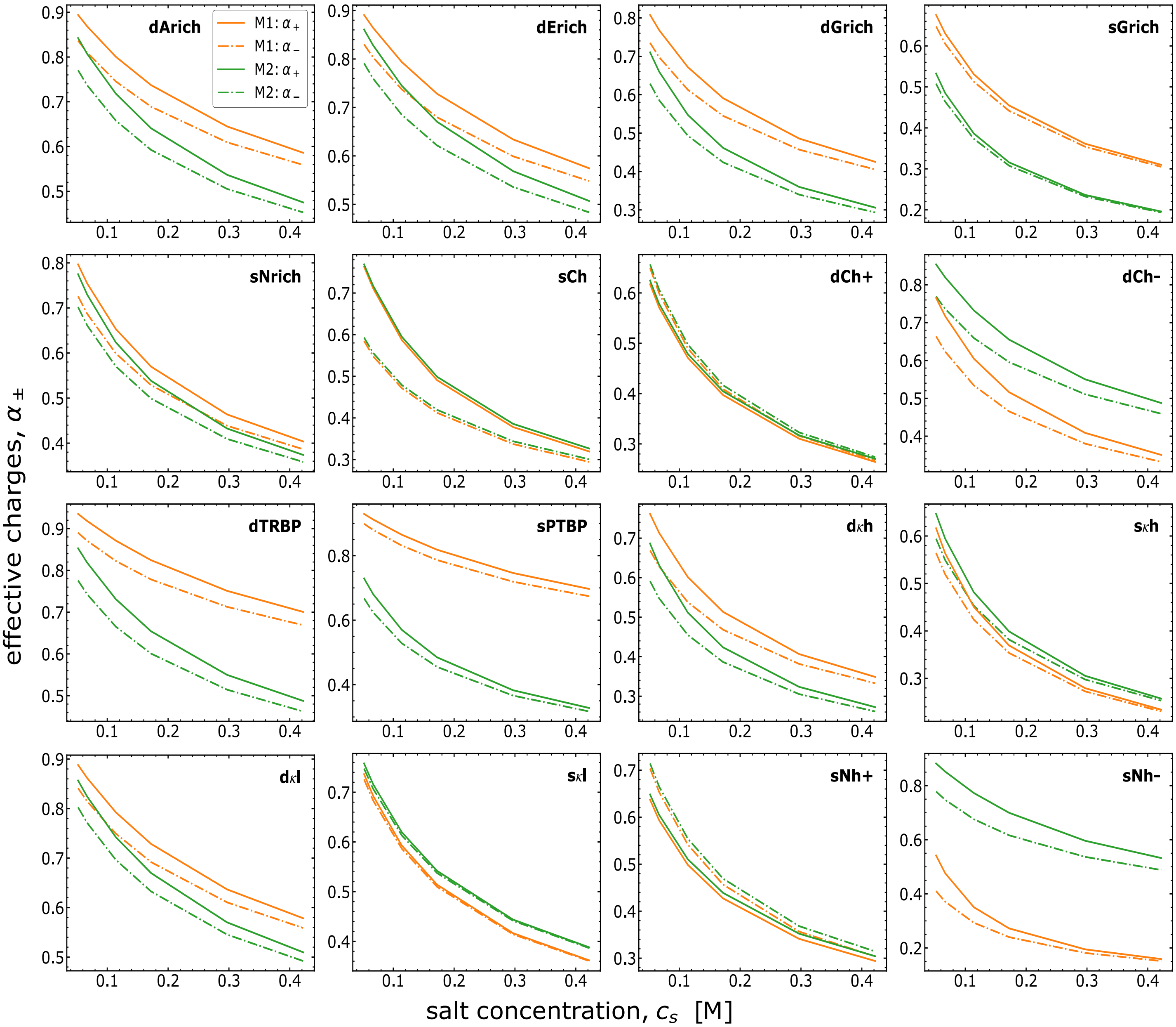

Figure S3: Effective charge (degrees of ionization) as a function of salt concentration (solid lines for positive charge, dashed lines for negative charge) for linker sequences predicted using parameters (of model M1, and near-predictive model M2) obtained by fitting data collected using Alexa 488/594 fluorophores. Charge can vary across a wide range, for each sequence and across sequences, but in all cases, ionization decreases as salt is increased because more ions are made available for condensation on charged groups in the IDR. The differences between the two models (M1 and M2), as seen for some sequences, are due to differences in the δ values (see Table S2)

The figure below shows the differences between fitted R_{ee} for all models (H-J, M0, M1, M2) and R_{ee} from the data collected using Alexa dyes.

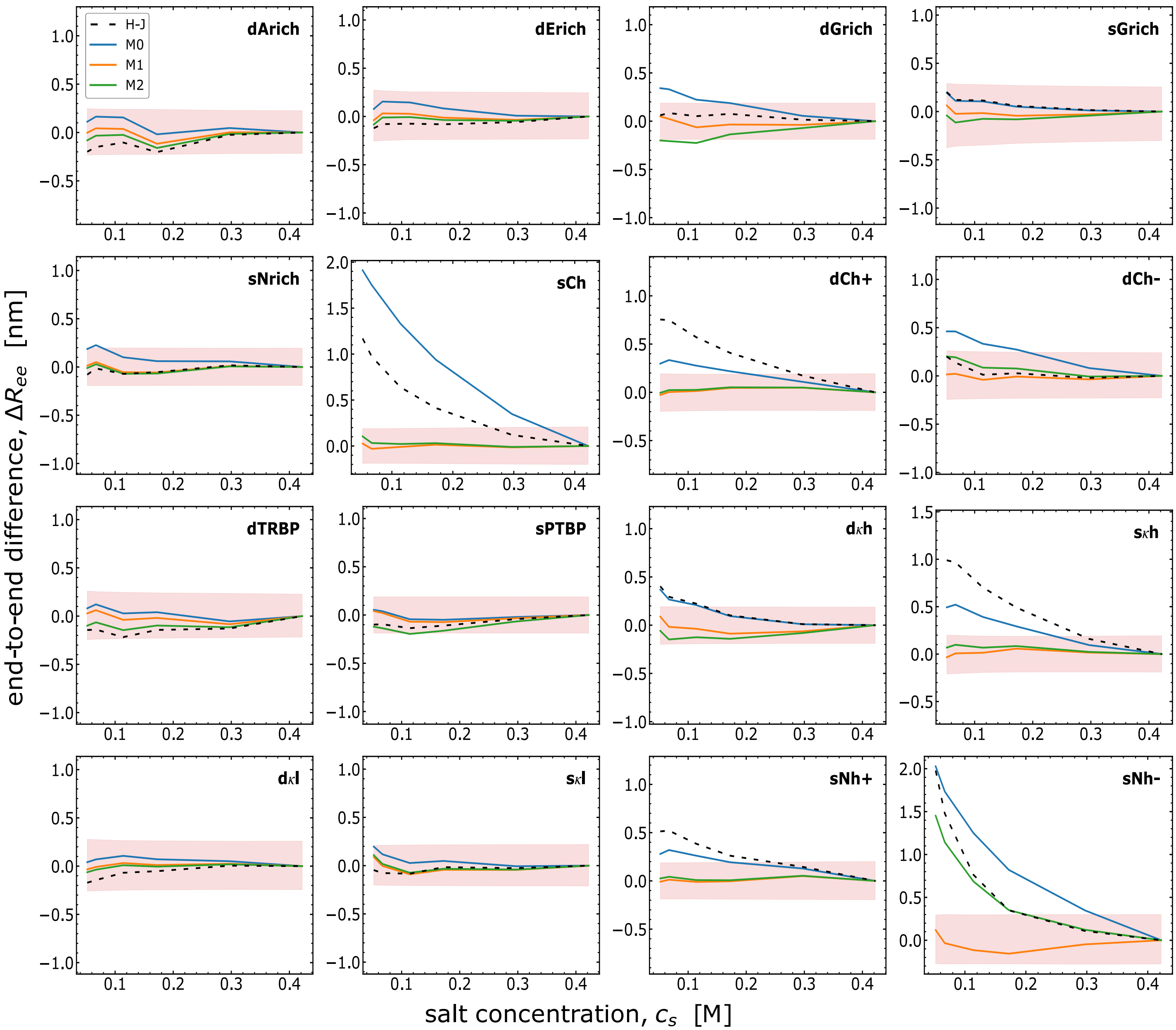

Figure S4: Differences between the end-to-end distances for linker sequences from all four models (H-J, M0, M1, M2) and end-to-end distances from the measurements show that counterion models (M1, M2) outperform simpler models (H-J, M0). These results are for Alexa 488/594 fluorophores. The shaded region indicates the systematic uncertainty in the single-molecule FRET measurements.

The figure below shows the mean errors (chi-squared), for each of the 16 linker IDRs labeled with the Alexa dyes, and the overall mean across all sequences.

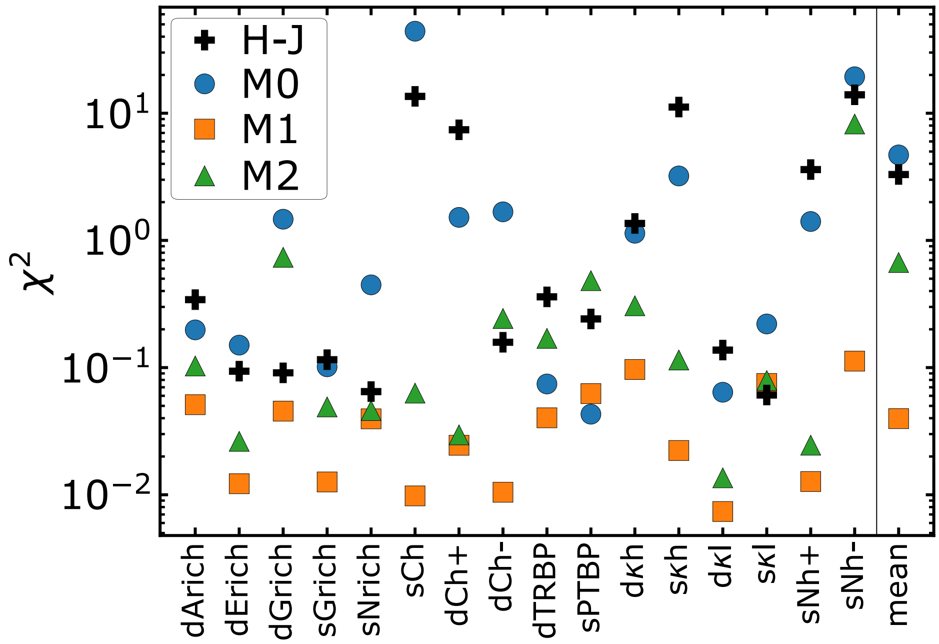

Figure S5: Chi-squared errors compared for the four models (H-J, M0, M1, M2) for each IDR with the Alexa dyes, with the overall mean across all sequences in the last entry, showing that counterion models (M1, M2) perform better than the simpler models (H-J, M0). A log scale is used to highlight the differences between models.

Here we compare the different components of the Free Energy contributions for two select sequences using parameters of model M1 inferred from data collected with the Alexa 488/594 fluorophores. For both sequences, fluctuation and entropic contributions $(F_1 + F_2 + F_3)$ increase with salt as expected, while free energy of ion pair formation (F_4) becomes more attractive due to the enhanced propensity to condense with salt. However, contrasting trends between dCh- and sκh are seen when the salt dependence of individual terms of the chain free energy (F_5) are computed. For polyelectrolyte-like sequences such as dCh-, charge-charge interaction is repulsive and decreases with increasing salt concentration due to screening, while sκh exhibits polyampholytic features with charge-charge attraction that decreases (becomes less negative) with increasing salt concentration. The total chain free energy (F_5) for dCh- is monotonically decreasing with salt, primarily due to screening of charge-charge interactions, which dominate over dipolar interactions, while sκh shows the opposite trend, since both charge-charge and charge-dipole interactions are attractive and get progressively screened with increasing salt concentration.

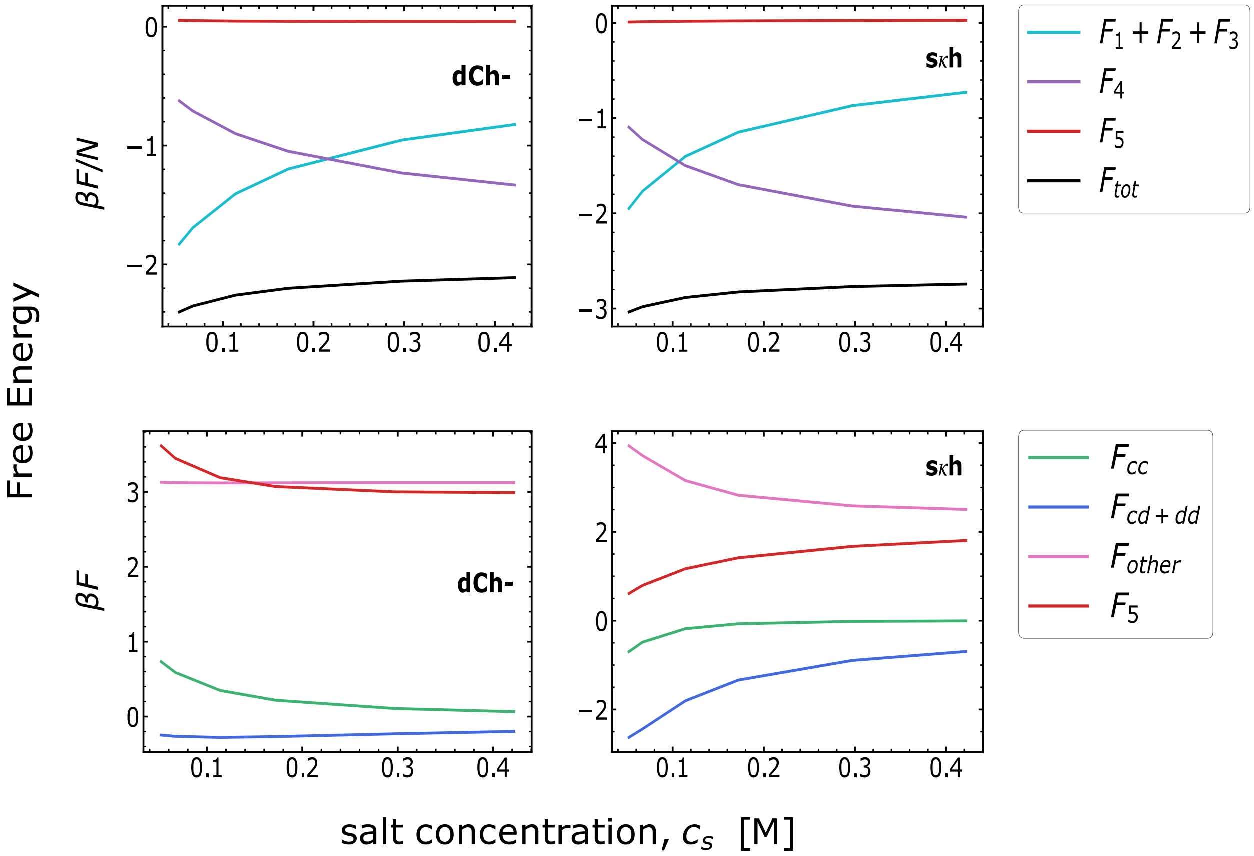

Figure S6: Free Energy contributions are compared as a function of salt concentration under model M1, for two select linker IDRs. The choice of sequences is based on composition: dCh$-$ (left) is polyelectrolyte-like, while sκh (right) is polyampholyte-like. The top row shows different contributions to the overall Free Energy terms: entropy and fluctuation contributions together $(F_1 + F_2 + F_3)$, ion pair formation (F_4), chain conformations and electrostatics (F_5), and the total (F_{tot}). These are evaluated in dimensionless units per monomer $(\beta F/N)$. The bottom row shows the different contributions of F_5: charge-charge electrostatics (F_{cc}), charge-dipole and dipole-dipole electrostatics (F_{cd+dd}), remaining contributions including non-electrostatic interactions (F_{other}), and total F_5. These are also dimensionless but for the entire chain (βF). Note that entropic and fluctuation terms F_2 and F_3 shown here are relative to the zero ionization case, i.e. $F_2 \to F_2(\alpha_\pm, c_s) - F_2(\alpha_\pm = 0, c_s)$ [and similar for F_3], because the salt contributions dominate; subtracting that part exposes the chain contributions.

The figure below is a consistency check to ensure that the M3 model (salt-dependent dielectric constant) compares well against the data collected using the Alexa dyes. However, this model is not of practical use since it requires knowing the value of the chain dimensions a priori.

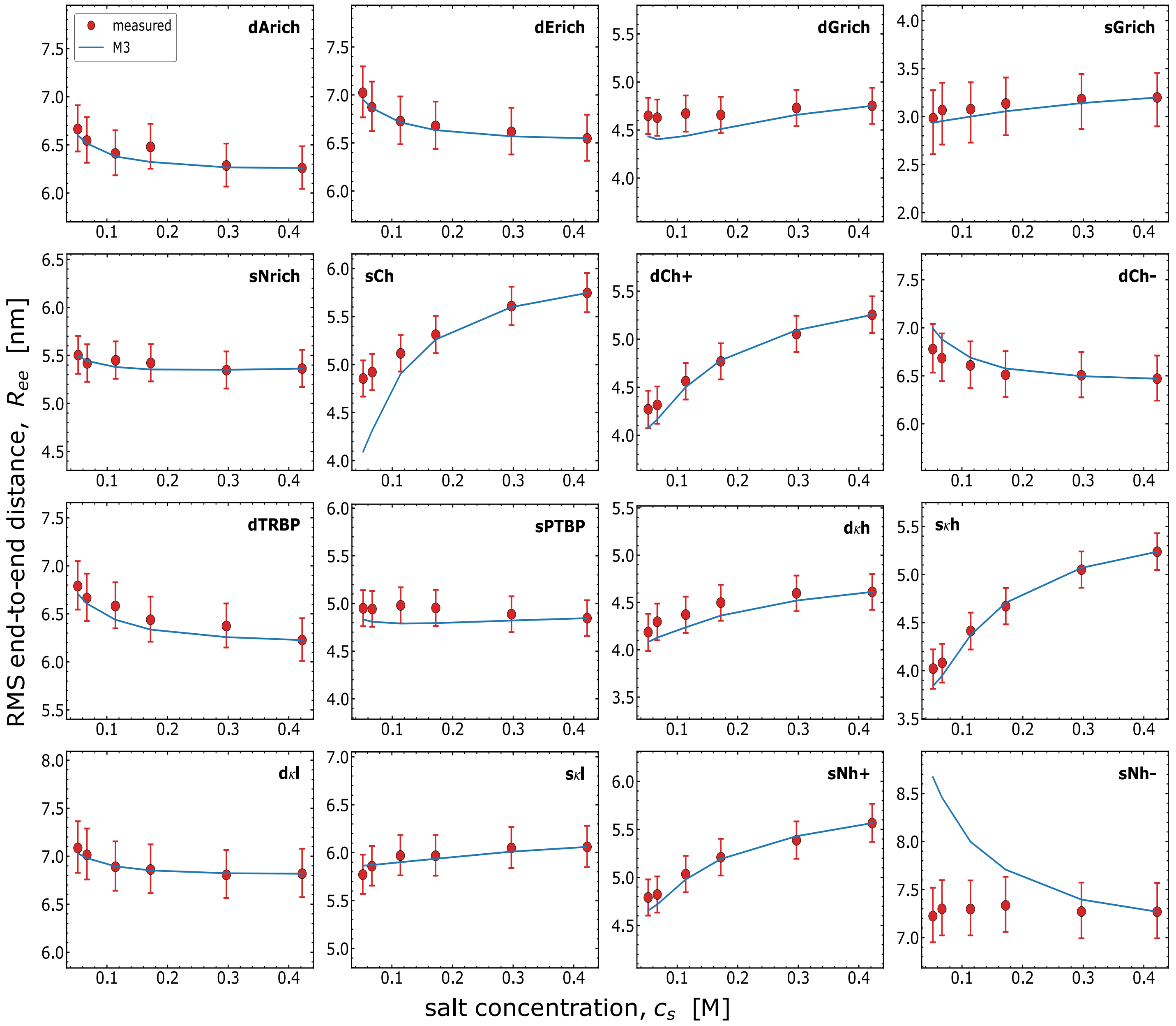

Figure S7: End-to-end distances as a function of salt concentration for all IDRs sequences labeled with Alexa 488/594, with the salt-dependent δ model (M3), based upon the linear fit (main text equation 24), compare well with FRET measurements. Model M3 performs slightly worse than M2, with χ^2 (excluding sNh−) of 0.513 for M3 and 0.166 for M2 (see main text Figure 2 and Table 1).

The figure below shows the predicted degrees of ionization from models M1 and M2 using parameters inferred by fitting the linker IDR data collected using the Cy3B/CF660R dye pair.

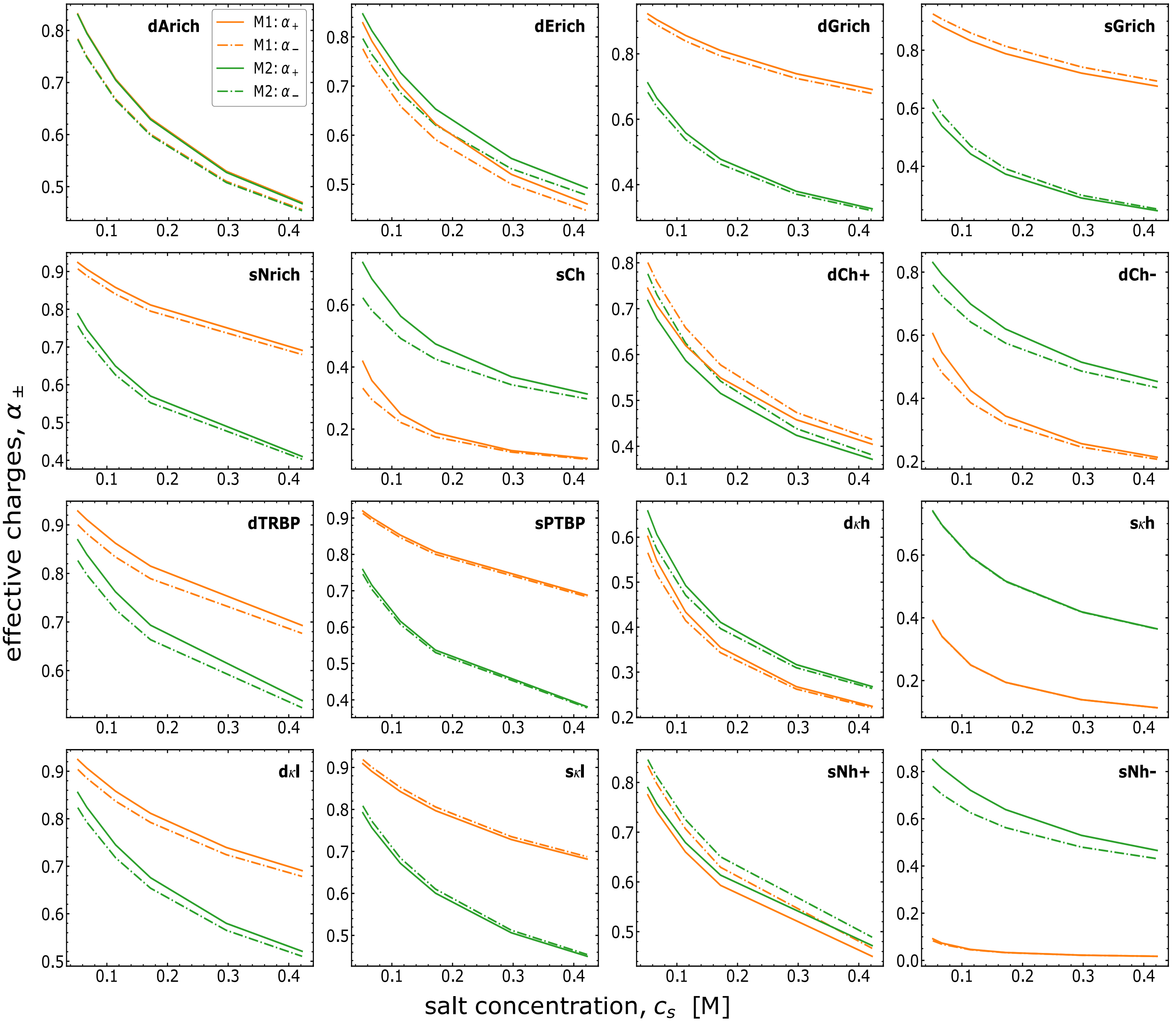

Figure S8: Effective charge (degree of ionization) as a function of salt concentration (solid lines for positive charge, dashed lines for negative charge) for linker IDRs predicted using parameters (of model M1, and near-predictive model M2) obtained by fitting data collected using the Cy3B/CF660R dye pair. As with the Alexa dyes (Figure S3), charge can vary across a wide range, for each sequence and across sequences, but in all cases, ionization decreases as salt is increased because more ions are made available for condensation on the charged groups of the IDR. The differences between two models (M1 and M2), as seen for some sequences, are due to differences in the δ values (see Table S3).

The figure below shows the differences between the fitted R_{ee} for all models (H-J, M0, M1, M2) and R_{ee} from linker IDR data collected using Cy3B/CF660R dye pair.

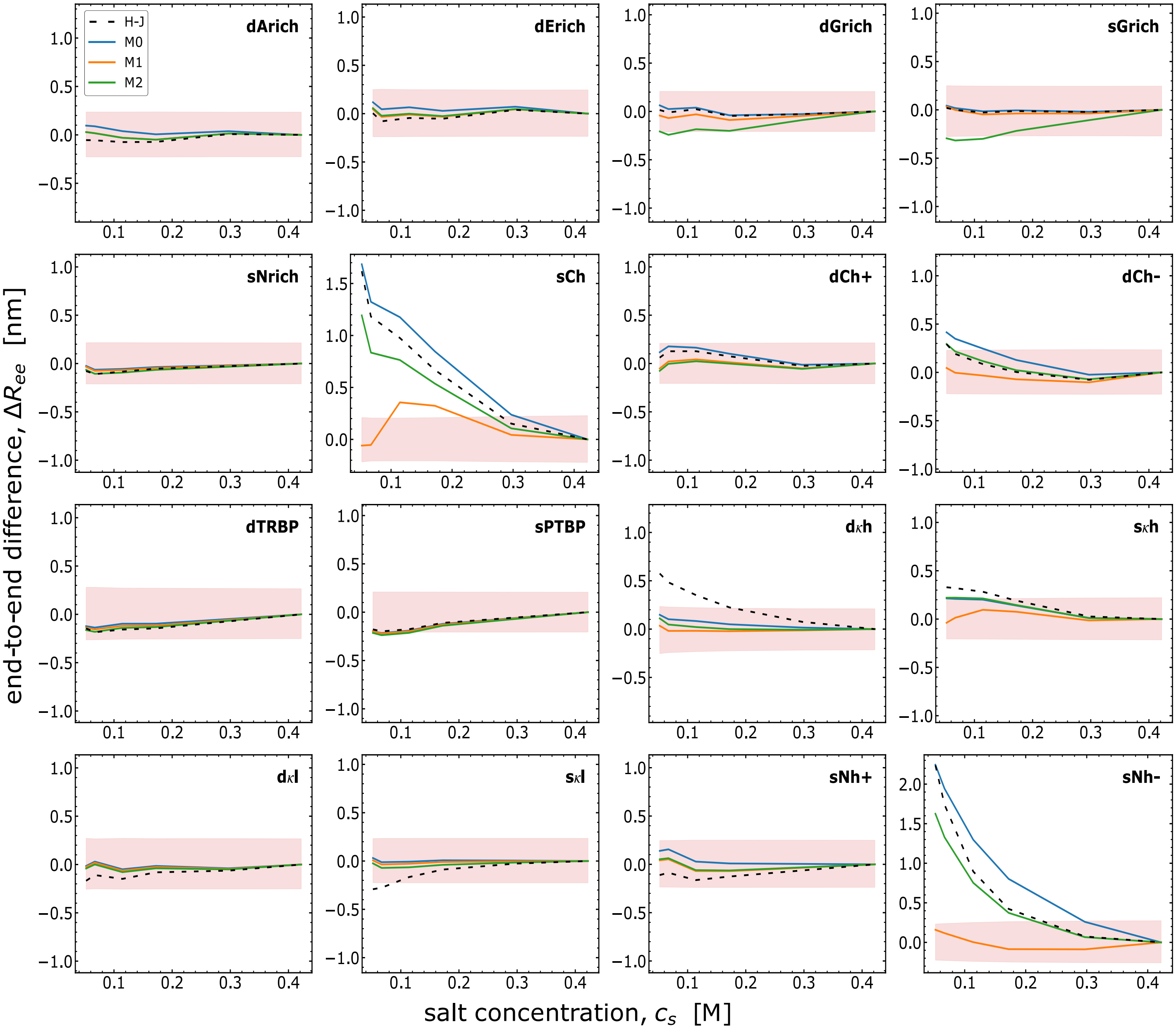

Figure S9: Differences between the end-to-end distances for linker IDRs from all four models (H-J, M0, M1, M2) and end-to-end distances from the measurements show that counterion models (M1, M2) outperform simpler models (H-J, M0). These results are for Cy3B/CF660R fluorophores. The shaded region indicates the span of error bars.

The figure below shows mean errors (chi-square), for each of the 16 IDRs with Cy3B/CF660R dyes, and the overall mean across sequences.

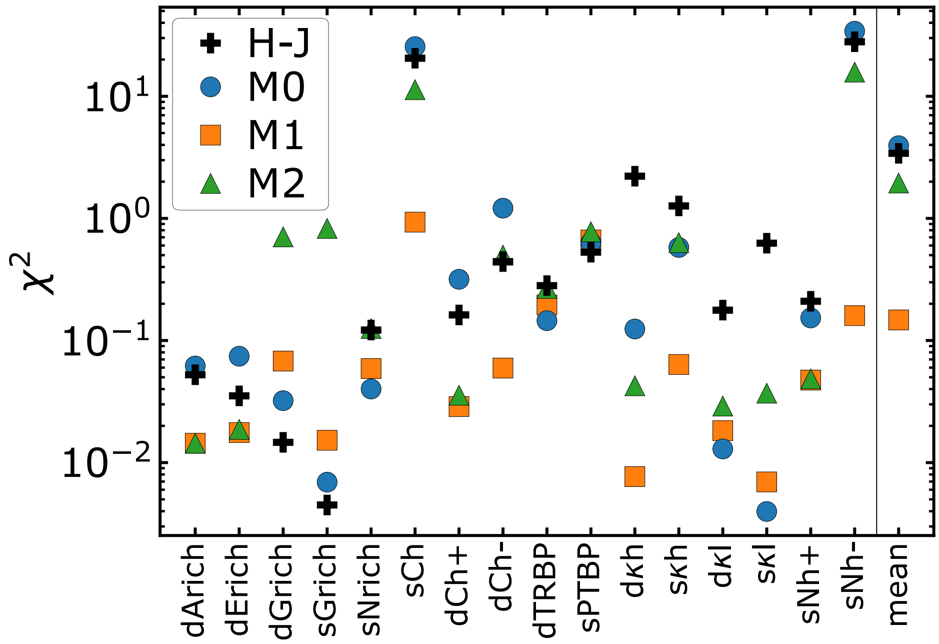

Figure S10: Chi-square errors visually compare the four models H-J, M0, M1, M2 for each IDR with Cy3B/CF660R dyes, with the overall mean across all sequences in the last entry, showing clearly that counterion models (M1, M2) perform better than simpler models (H-J, M0) for most sequences. A log scale is used to highlight the differences between models.

The figure below is a consistency check to ensure that the M3 model (salt-dependent dielectric constant) compares well with data on the linker IDRs collected using the Cy3B/CF660R dye pair.

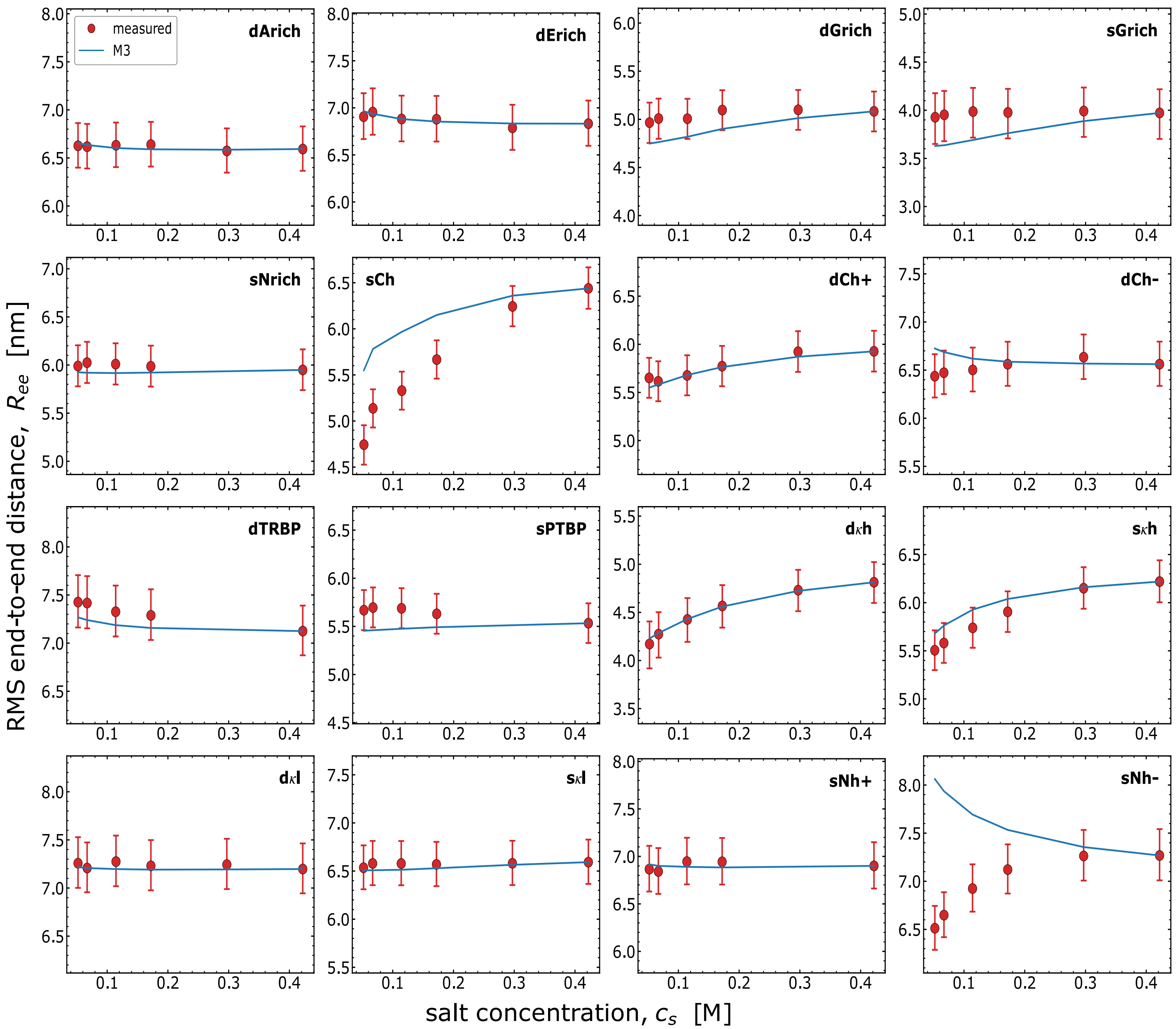

Figure S11: End-to-end distances as a function of salt concentration for all linker IDRs labeled with Cy3B/CF660R, under the salt-dependent δ model (M3), based upon the linear fit (main text equation 24), compare well with FRET measurements. Model M3 performs slightly better than M2, with χ^2 (excluding sNh−) of 0.691 and 1.026, respectively (see main text Figure 4 and Table 2).

Normalized distance maps d^*_{ij} predicted from model M1 and M0 using parameters inferred by fitting data collected for the linker IDRs using the Cy3B/CF660R dye pair.

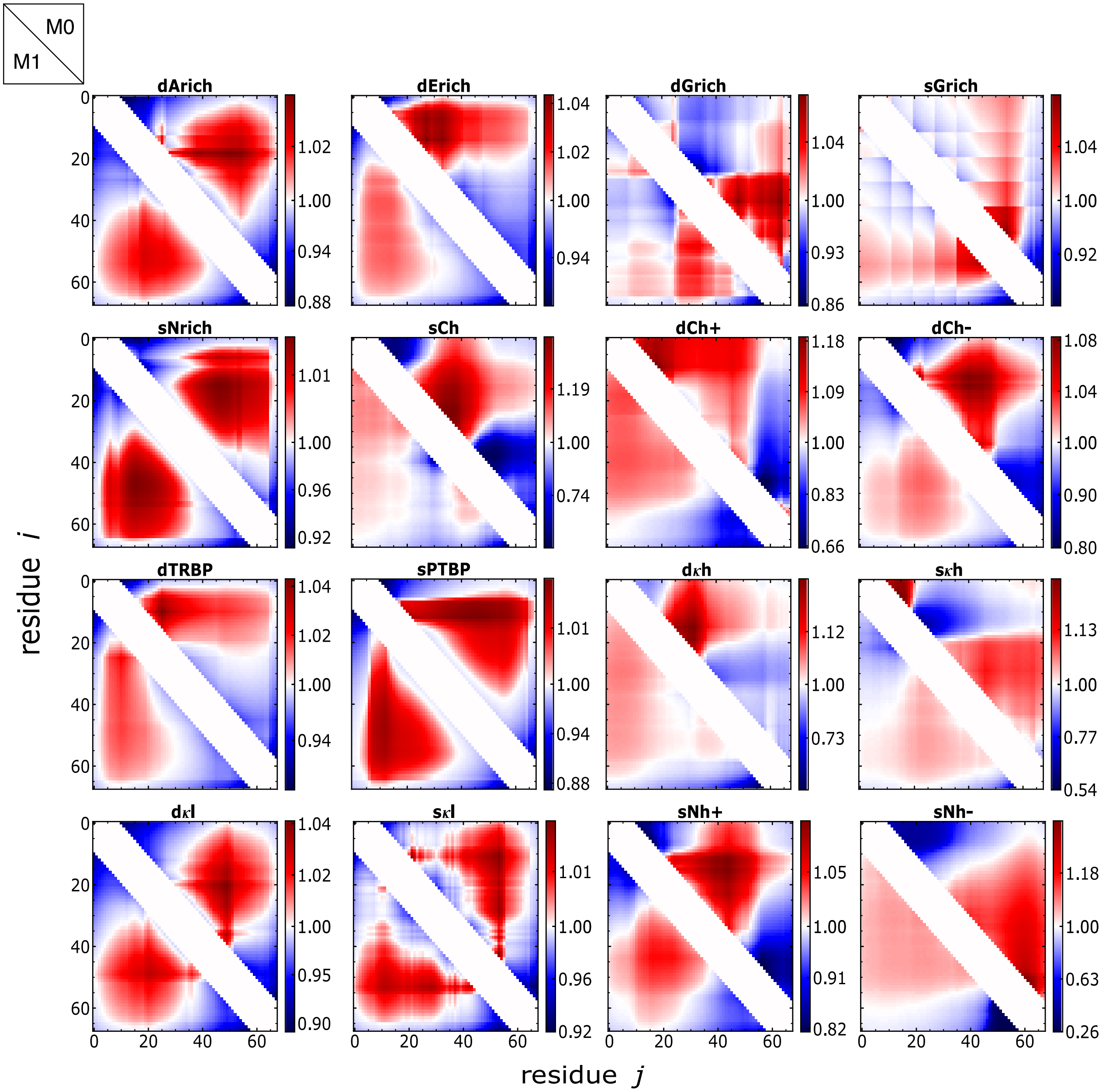

Figure S12: Inter-residue distance maps (normalized by fits to homopolymer scaling) for the linker IDRs under near-physiological conditions (150 mM salt and $T = 20\,°C$), predicted using parameters (determined by matching end-to-end distance data collected using the Cy3B/CF660R dye pair) of model M1 (bottom triangle), and model M0 (upper triangle).

Normalized distance maps d^*_{ij} for the linker IDRs predicted from model M1 compared between the dye pairs Alexa488/594 and Cy3B/CF660R.

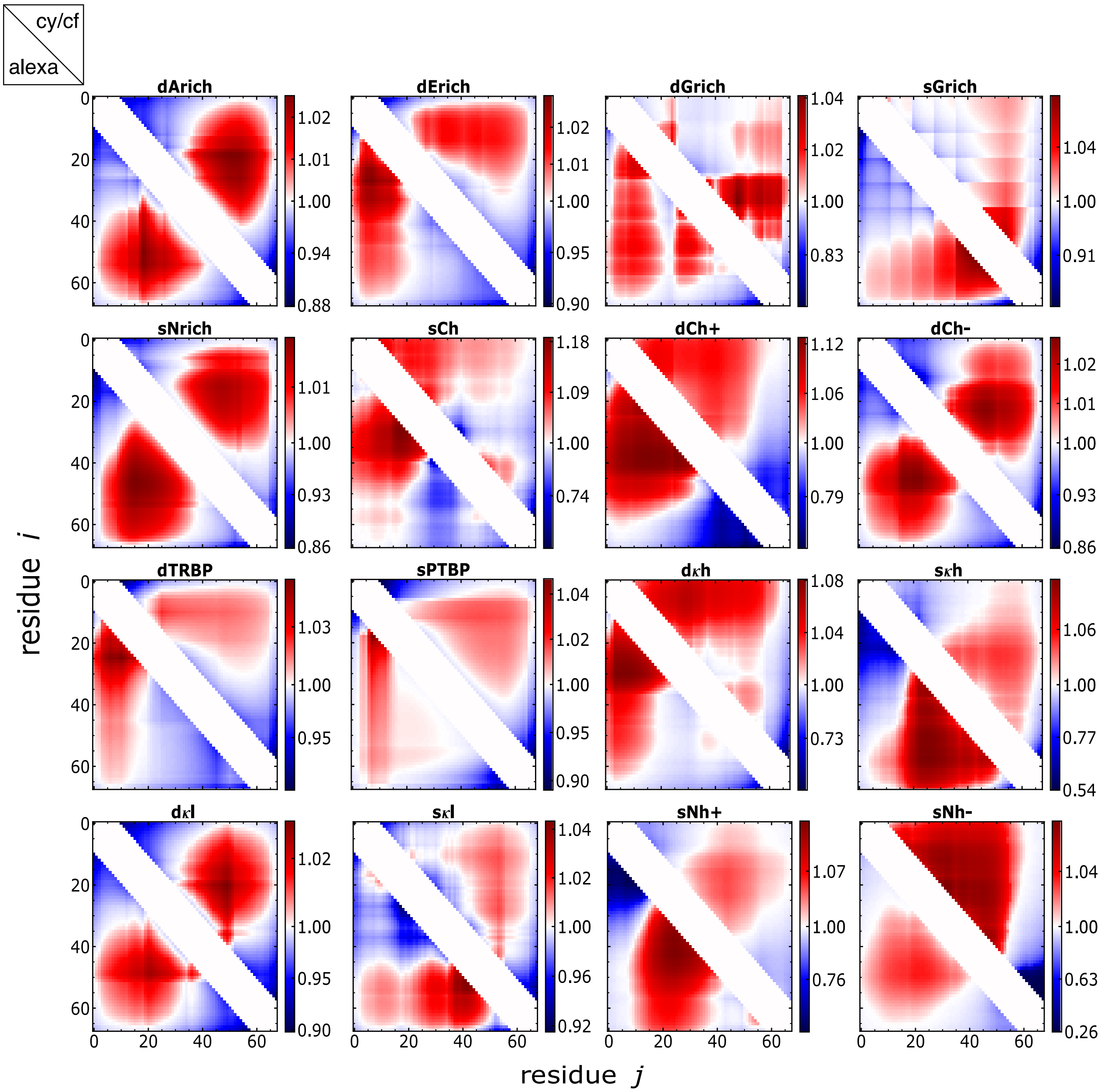

Figure S13: Inter-residue distance maps (normalized by fits to homopolymer scaling) for the linker IDRs under near-physiological conditions (150 mM salt and $T = 20\,°C$), predicted using parameters of model M1 (see Tables S2 and S3) for Alexa488/594 (bottom triangle), and Cy3B/CF660R (upper triangle).

The figure below displays predicted effective charges (degrees of ionization) associated with the prediction of distance between a specific residue pair (segments) of Stm and ProTα.

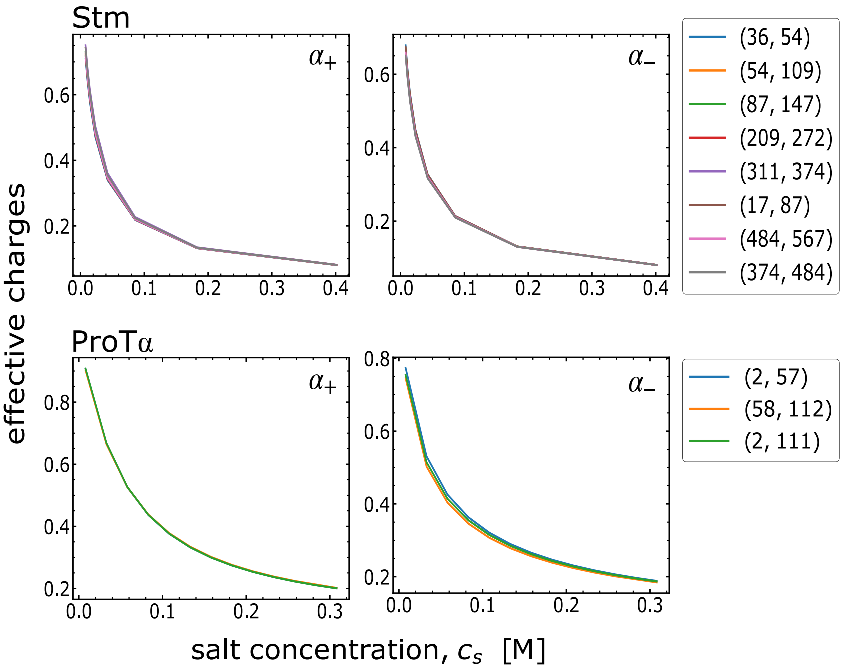

Figure S14: Predicted effective charges (degrees of ionization), for positive and negative charges (α_+, α_-), when free energy is minimized to determine the distance between a specific residue pair (segments) for Stm and ProTα as a function of salt concentration (c_s). These values vary little between different residue pairs. Legends are labeled by residue pairs, (i, j), for each sequence, although curves are difficult to distinguish due to their similarity. These results are not enforced by construction, but are emergent from the model, indicating that the counter-ion theory predicts highly similar results for charges across pairs/segments, even if x_{ij} (and R_{ij}) are distinct between pairs/segments. These results correspond to main text Figure 6, and were obtained self-consistently in the same calculation.

The figure below shows the comparison between inter-residue distances R_{ij} for the linker IDRs predicted from M2 model and the $C_\alpha - C_\alpha$ distance, between the pair of residues $(i, j) = (5, 63)$, measured using the Alexa dye pair.

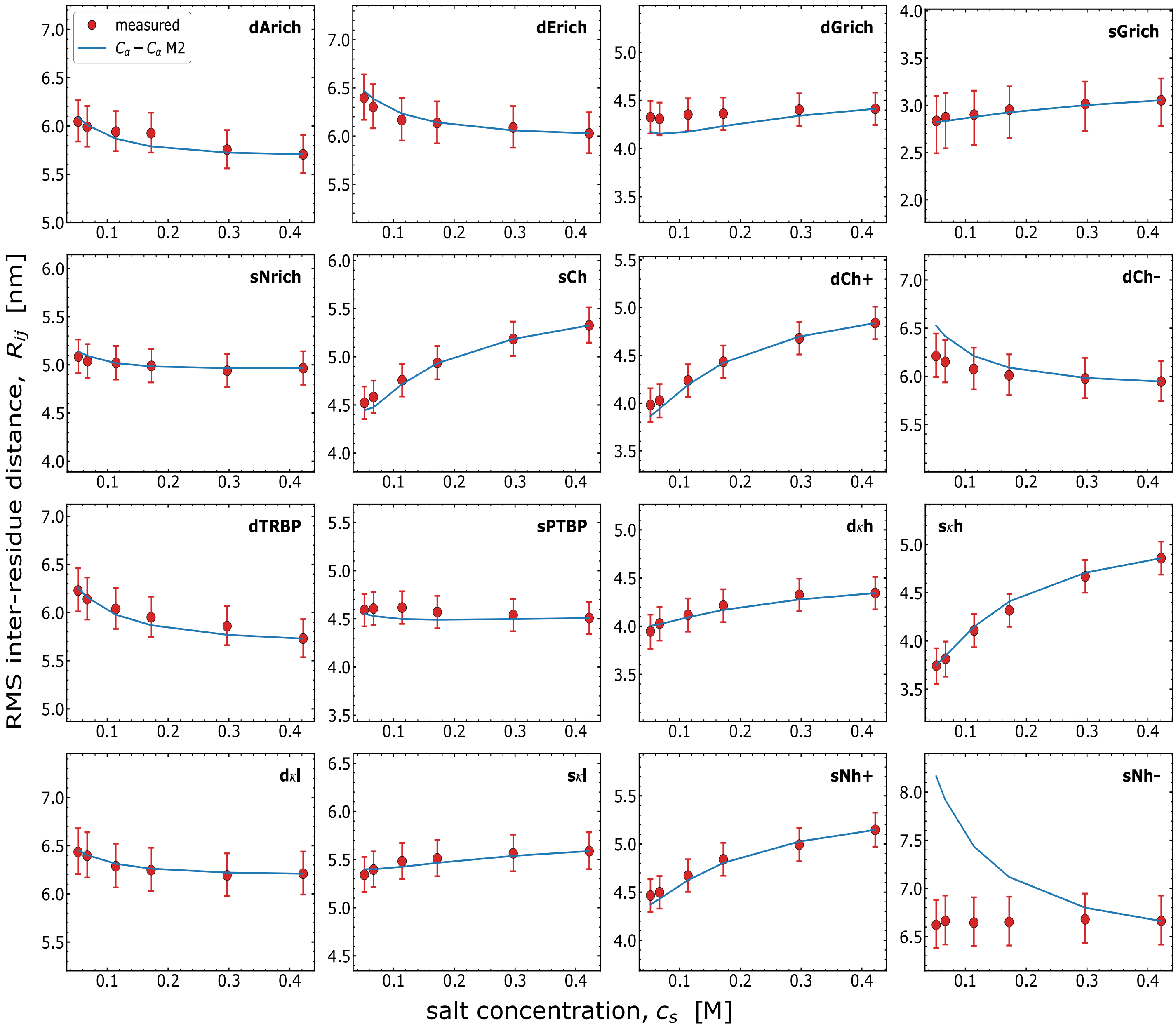

Figure S15: Inter-residue distances ($C_\alpha - C_\alpha$) as a function of salt concentration for all linker IDRs labeled with Alexa 488/594, modeled using M2, compare well with FRET measurements. This differs from results in main text Figure 2 in three ways: $C_\alpha - C_\alpha$ distances were inferred from FRET efficiencies, dyes were modeled differently (see Sec. S3), and the underlying theory for R_{ij} with residue pair $(i, j) = (5, 63)$ was used (see main text Sec. 2.2 and 3.5). $C_\alpha - C_\alpha$ based on M2 performs similarly to end-to-end M2, with χ^2 (excluding sNh−) of 0.147 and 0.166, respectively (see main text Figure 2 and Table 1).

The figure below shows the comparison between inter-residue distances R_{ij} for the linker IDRs predicted from the M2 model and the $C_\alpha - C_\alpha$ distance, between the pair of residues $(i,j) = (5, 63)$, measured using the Cy3B/CF660R dye pair.

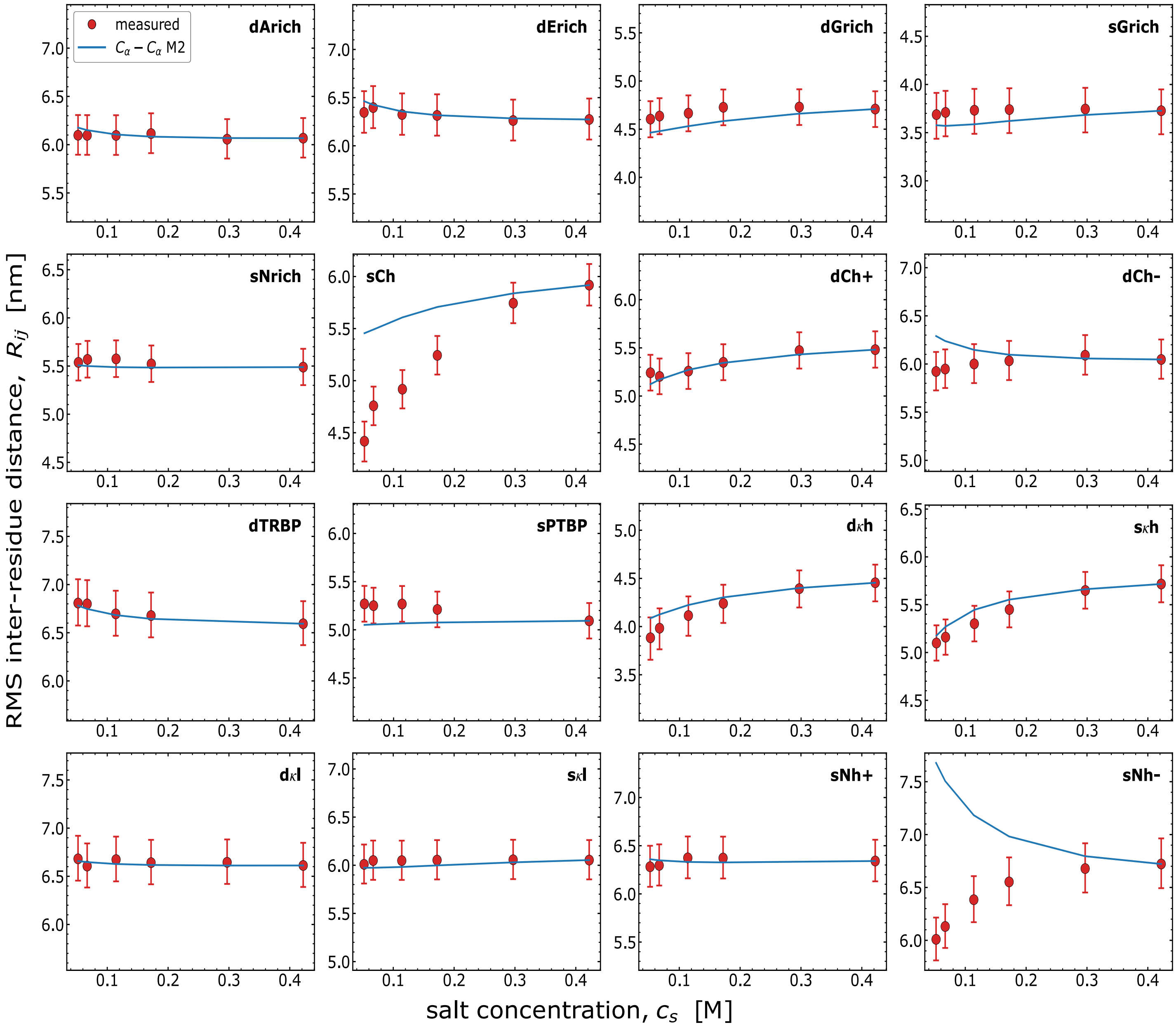

Figure S16: Inter-residue distances ($C_\alpha - C_\alpha$) as a function of salt concentration for all linker IDRs labeled with Cy3B/CF660R, modeled using M2, compare well with FRET measurements. This differs from results in the main text Figure 4 in three ways: $C_\alpha - C_\alpha$ distances were inferred from FRET efficiencies, dyes were modeled differently (see Sec. S3), and the underlying theory for R_{ij} with residue pair $(i,j) = (5, 63)$ was used (see main text Sec. 2.2 and 3.5). $C_\alpha - C_\alpha$ based on M2 performs similarly to end-to-end M2, with χ^2 (excluding sNh−) of 0.956 and 1.026, respectively (see main text Figure 4 and Table 2).

References

[1] Andrea Holla, Erik W. Martin, Thomas Dannenhoffer-Lafage, Kiersten M. Ruff, Sebastian L. B. König, Mark F. Nüesch, Aritra Chowdhury, John M. Louis, Andrea Soranno, Daniel Nettels, Rohit V. Pappu, Robert B. Best, Tanja Mittag, and Benjamin Schuler. Identifying sequence effects on chain dimensions

of disordered proteins by integrating experiments and simulations. *JACS Au*, 4(12):4729–4743, 2024. doi: 10.1021/jacsau.4c00673. URL https://doi.org/10.1021/jacsau.4c00673.

[2] J. K. Ko and J. Ma. A rapid and efficient pcr-based mutagenesis method applicable to cell physiology study. *Am. J. Physiol. Cell Physiol.*, 288(6):C1273–8, 2005. ISSN 0363-6143 (Print) 0363-6143 (Linking). doi: 10.1152/ajpcell.00517.2004. URL https://www.ncbi.nlm.nih.gov/pubmed/15659713.

[3] Tomasz M. Kapłon, Grzegorz Rymarczyk, Małgorzata Nocula-Ługowska, Michał Jakób, Marian Kochman, Marek Lisowski, Zbigniew Szewczuk, and Andrzej Ożyhar. Starmaker exhibits properties of an intrinsically disordered protein. *Biomacromolecules*, 9(8):2118–2125, 2008. doi: 10.1021/bm800135m. URL https://doi.org/10.1021/bm800135m.

[4] A Borgia, M B Borgia, K Bugge, V M Kissling, P O Heidarsson, C B Fernandes, A Sottini, A Soranno, K J Beholder, D Nettles, B B Kragelund, R B Best, and B Schuler. Extreme disordered in an ultrahigh-affinity protein complex. *Nature*, 555:61–66, 2018.

[5] A Chowdhury, A Borgia, S Ghosh, A Sottini, S Mitra, R.S Eapan, MB Borgia, T Yang, N Galvanetto, M Ivanovic, P Lukijanczuk, R Zhu, D Nettels, A Kundagrami, and B Schuler. Driving forces of the complex formation between highly charged disordered proteins. *Proc. Natl. Acad. Sci.*, 120(41): e2304036120, 2023.

[6] Volodymyr Kudryavtsev, Martin Sikor, Stanislav Kalinin, Dejana Mokranjac, Claus A. M. Seidel, and Don C. Lamb. Combining MFD and PIE for accurate single-pair förster resonance energy transfer measurements. *ChemPhysChem*, 13(4):1060–1078, 2012. doi: 10.1002/cphc.201100822. URL https://doi.org/10.1002/cphc.201100822.

[7] B. Schuler. Application of single molecule förster resonance energy transfer to protein folding. *Methods Mol. Biol.*, 350:115–38, 2007. URL http://www.ncbi.nlm.nih.gov/entrez/query.fcgi?cmd=Retrieve&db=PubMed&dopt=Citation&list_uids=16957321.

[8] Erik D. Holmstrom, Andrea Holla, Wenwei Zheng, Daniel Nettels, Robert B. Best, and Benjamin Schuler. *Accurate Transfer Efficiencies, Distance Distributions, and Ensembles of Unfolded and Intrinsically Disordered Proteins From Single-Molecule FRET*, pages 287–325. Elsevier, 2018. doi: 10.1016/bs.mie.2018.09.030. URL https://doi.org/10.1016/bs.mie.2018.09.030.

[9] Nam Ki Lee, Achillefs N. Kapanidis, You Wang, Xavier Michalet, Jayanta Mukhopadhyay, Richard H. Ebright, and Shimon Weiss. Accurate FRET measurements within single diffusing biomolecules using alternating-laser excitation. *Biophysical Journal*, 88(4):2939–2953, 2005. doi: 10.1529/biophysj.104.054114. URL https://doi.org/10.1529/biophysj.104.054114.

[10] Matthew Antonik, Suren Felekyan, Alexander Gaiduk, and Claus A. M. Seidel. Separating structural heterogeneities from stochastic variations in fluorescence resonance energy transfer distributions via photon distribution analysis. *The Journal of Physical Chemistry B*, 110(13):6970–6978, 2006. doi: 10.1021/jp057257. URL https://doi.org/10.1021/jp057257.

[11] Patrick Ernst, Franziska Zosel, Christian Reichen, Daniel Nettels, Benjamin Schuler, and Andreas Plückthun. Structure-guided design of a peptide lock for modular peptide binders. *ACS Chemical Biology*, 15(2):457–468, 2020. doi: 10.1021/acschembio.9b00928. URL https://doi.org/10.1021/acschembio.9b00928.

[12] W Zheng, G H Zerze, A Borgia, J Mittal, B Schuler, and R B Best. Inferring properties of disordered chains from fret transfer efficiencies. *Journal of Chemical Physics*, 148:123329, 2018.

[13] H Hofmann, A Soranno, A Borgia, K Gast, D Nettels, and B Schuler. Polymer scaling laws of unfolded and intrinsically disordered proteins quantified with single-molecule spectroscopy. *Proc. Natl. Acad. Sci.*, 109(40):16155–16160, 2012.

[14] PG Higgs and JF Joanny. Theory of polyampholyte solutions. *J. Chem. Phys.*, 94:1543–1554, 1991.

[15] E W Martin, A S Holehouse, C R Grace, A Hughes, R V Pappu, and T Mittag. Sequence determinants of the conformational properties of an intrinsically disordered protein prior to and upon multisite phosphorylation. *J. Am. Chem. Soc.*, 138:15323–15335, 2016.

[16] J Huihui and K Ghosh. An analytical theory to describe sequence-specific inter-residue distance profiles for polyampholytes and intrinsically disordered proteins. *J. Chem. Phys.*, 52:161102, 2020.

[17] NIST computational chemistry comparison and benchmark database. `http://cccbdb.nist.gov/`, 2022. URL `http://cccbdb.nist.gov/`.

[18] M Phillips, M Muthukumar, and K Ghosh. Beyond monopole electrostatics in regulating conformations of intrinsically disordered proteins. *PNAS Nexus*, 3(9):367, 2024.

[19] T J Nott, E Petsalaki, P Farber, D Jervis, E Fussner, A Plochowietz, T D Craggs, D P Bazett-Jones, T Pawson, J D Forman-Kay, and AJ Baldwin. Phase transition of a disordered nuage protein generates environmentally responsive membraneless organelles. *Molecular Cell*, 57(5):936–947, 2015.

[20] T Firman and K Ghosh. Sequence charge decoration dictates coil-globule transition in intrinsically disordered proteins. *J. Chem. Phys.*, 148(12):123305, 2018.

[21] L Houston, M Phillips, A Torres, K Gaalswyk, and K Ghosh. Physics-based machine learning trains hamiltonians and decodes sequence-conformation relation in the disordered proteome. *Journal of Chemical Theory and Computation*, 20(22):10266–10274, 2024.

[22] J Huihui, T Firman, and K Ghosh. Modulating charge patterning and ionic strength as a strategy to induce conformational changes in intrinsically disordered proteins. *J. Chem. Phys.*, 149:085101, 2018.
